# Examining children’s sensitivity to morphologically-marked focus in the comprehension of question sentences: evidence from quantifier comprehension in child Turkish

**DOI:** 10.3389/fnhum.2026.1914200

**Published:** 2026-10-14

**Authors:** Münir Özturhan, Utako Minai

**Affiliations:** Department of Linguistics, University of Kansas, Lawrence, KS, United States

**Keywords:** child language comprehension, focus marking, morphological focus, quantifier comprehension, Turkish

## Abstract

This study investigated the role of morphologically-marked focus in child language comprehension. The Turkish question particle (QP) *mI* typically appears at the end of a question sentence, but can also appear in other positions, attaching to an element to be focalized and marking it as the most important information conveyed in the sentence. For example, if a question sentence meaning “*Is the child holding a balloon?*” has the QP on the subject noun (“*the child*”), it is focalized, rendering an interpretation similar to a cleft construction (e.g., “*Is it the child that is holding a balloon?*”). We investigated whether Turkish-speaking preschool-age children can incorporate focus marked by QP placement by testing sentences containing a universal quantifier *her* (“every”) in two conditions: questions with the QP appearing sentence-finally (yielding a reading like “*Is every child holding a balloon?*”), and those with the QP on the universally-quantified subject, focalizing it (yielding an “*Is it every child that is holding a balloon?*” reading). We presented the sentences along with an “extra object” picture depicting children holding a balloon each and an extra balloon held by nobody, a context known to elicit “q-spreading” (erroneous “No” answers attributed to the presence of the extra balloon), to examine whether focus-marking on the subject would facilitate adult-like interpretation (“Yes” answers verifying the children all have balloons). Children indeed exhibited significantly less q-spreading when the quantified subject contained focus-marking, suggesting that children utilize morphologically-marked focus during comprehension, consistent with “full competence” approaches to focus and quantification.

## 1 Introduction

Languages deploy numerous ways to manipulate focus, marking an element in a sentence which conveys the central and most important information (that which is focused/focalized) as compared to the rest of the conveyed information (that which is presupposed). Focus marking is implemented via multiple linguistic devices including phonological, prosodic and intonational realization of focus (e.g., Ladd, 2008; Pierrehumbert and Hirschberg, 1990; Selkirk, 1995), using focus-sensitive elements such as focus particles (e.g., *only*, *also*, *even* in English) (e.g., Kadmon, 2001; Rooth, 1992, 1996), or syntactic movement such as clefting (e.g., Chomsky, 1977). Focus is a crucial aspect of semantic/pragmatic comprehension, as focus can provide the comprehender with cues to utilize in arriving at an accurate and pragmatically felicitous interpretation of an utterance.

In the experimental literature on how focus is processed and comprehended by adult speakers, findings suggest that adult speakers utilize focus in a range of contexts. For example, focus serves as a cue to resolve syntactic ambiguity (e.g., Schafer, 1996; Sedivy, 2002), to resolve reference (e.g., Dahan et al., 2022; Kurumada et al., 2014; Weber et al., 2006), and to derive detailed pragmatic meanings incorporating a question under discussion that varies from discourse to discourse (e.g., Clifton and Frazier, 2012; Cummins and Rohde, 2015). However, the extent to which *young children*, particularly those in early childhood (aged 3–6 years old) are sensitive to different linguistic realizations of focus in different languages remains unclear, particularly as regards comprehension, with the existing evidence. In particular, the existing evidence regarding comprehension leads to opposing claims regarding whether children possess adult-like competence regarding focus. Moreover, some linguistic realizations of language (including morphologically-marked focus) have remained understudied, leaving the scope of children’s sensitivity to focus as a matter of debate (see, e.g., Szendrői et al., 2018 for discussion). This motivates the current study, which examines sensitivity to morphologically-marked focus in child Turkish.

Children’s acquisition of focus has been investigated primarily in two domains. The first is children’s comprehension of focus-sensitive elements, e.g., focus particles such as *only* in English. In this domain, children do not uniformly show adult-like sensitivity to focus particles, but rather show an asymmetry in which the comprehension of subject-associated *only* is less target-like than for object-associated *only*. For example, as regards *only* in English, sentences with subject-associated *only* (as in, *Only the cat is holding a balloon*) tend to be incorrectly interpreted by children as if the sentence contained *only* in the object/predicate (that is, they interpret *Only the cat is holding a balloon* on par with *The cat is only holding a balloon*). This non-adult-like comprehension pattern has been reported for children acquiring a range of languages, including English (Crain et al., 1992; Notley et al., 2009; Kim, 2011; Paterson et al., 2003), Japanese (Endo, 2004), Mandarin (Zhou and Crain, 2010), German (Höhle et al., 2016), European Portuguese (Santos, 2006) and Turkish (Topaloğlu and Nakipoğlu, 2017). The second domain of research regards children’s use of prosodically-marked focus. In production, children start to mark focus prosodically early, such that toddlers and preschoolers already modulate prosody to distinguish focused from unfocused constituents (e.g., Höhle et al., 2009b; Wells et al., 2004; Wonnacott and Watson, 2008). By around 4–5 years of age, children are adult-like in several aspects of prosodic focus marking across a range of languages, with the developmental trajectory mainly impacted by the typology of the target language (see Chen, 2018 for a review). In contrast, research on comprehension has yielded mixed results, with some studies suggesting that children face challenges in incorporating the meaning of prosodic focus as measured in both offline comprehension tasks (e.g., Wells et al., 2004) and in online measures such as eye-tracking (e.g., Ito et al., 2012; Sekerina and Trueswell, 2012; Tang et al., 2023).

The findings reviewed above contrast children’s early use of focus in production and their difficulties with focus in comprehension. This asymmetry has motivated two competing accounts of focus acquisition. “Partial competence” accounts hold that young children lack the fundamentally adult-like linguistic competence required for the comprehension of focus (e.g., Cutler and Swinney, 1987). “Full competence” accounts, in contrast, attribute their difficulties to factors that hinder access to a fully-intact competence (e.g., Höhle et al., 2009a; Szendrői et al., 2018). For example, one “full competence” account attributes children’s challenges to the alternatives evoked by focus. When focus is marked on the subject of a sentence, e.g., by means of the focus particle *only* (*Only the cat is holding a balloon*) or by modulating prosody placing an focal stress (*the CAT is holding a balloon*), it evokes a set of relative alternatives (e.g., a dog and a rabbit), and renders a “contrastive focus” interpretation such that, among the members of the set, the cat is the only balloon-holder, while the alternative members are not (Rooth, 1992, 1996). To arrive at such an interpretation, it is required to establish a contrastive set encompassing appropriate members considered in a given context and properly focalize the right member from among the alternatives, which may create computational challenges for children (e.g., Ito et al., 2012, 2014; Ito, 2014). However, it remains unclear whether children can demonstrate adult-like use of focus when it is expressed through means other than syntactic or prosodic operations used by different languages. It also remains unclear whether any difficulties children exhibit can be attributed to factors other than their knowledge of focus itself, as predicted by “full competence” accounts but not by “partial competence” accounts.

## 2 Attentional focus marking: Turkish as a test case

The current study examines the role of linguistic focus in child language by investigating children’s sensitivity to morphological focus-marking in Turkish that conveys *attentional focus* (Kim, 2019), such that the focalized information draws more robust attention than the presupposed information (e.g., Bredart and Modolo, 1988; Dodd and Bradshaw, 1980; Engelkamp and Zimmer, 1983; Sturt et al., 2004). The comprehension of attentional focus is difficult to study given that its contribution to meaning is relatively subtle, often not affecting the core semantic meaning of the sentence. However, in the literature on adult language processing, placing focus on a particular position in the sentence is thought to increase attention to those positions during processing, suggesting that at least in adult system the attention plays an important role (e.g., Hoeks et al., 2023; Husband and Ferreirra, 2016). One case utilized in the child language literature, where attentional focus is overtly marked, is clefting (e.g., *It is the cat that is holding a balloon*). Existing work suggests that accuracy in children’s comprehension of cleft sentences can vary depending on the syntactic status of the focalized element (subject vs. object) or the degree by which pragmatics supports the felicity of focalization (Aravind et al., 2018). These findings provide some evidence that children may have adult-like sensitivity to what is conveyed by clefting, despite facing challenges overall, to the extent that their comprehension improved with pragmatic support. However, the source of the difficulty that children face with clefting remains unclear. For example, it may reflect difficulty with the syntax of clefting, or difficulty in incorporating the focus information that clefting conveys into their overall interpretation of an utterance.

In light of this, the current study examined children’s sensitivity to attentional focus marking in Turkish, where attentional focus can be marked morphologically without the syntactic complexity of clefting (Atlamaz, 2023). This provides an ideal testing ground to examine whether children can utilize focus during comprehension when not required to compute an additional syntactic structure like a cleft to identify what was focused.

To illustrate the mechanism of morphologically-marked attentional focus in Turkish, let us first lay out some fundamental grammatical characteristics of Turkish sentences. Consider (1), exemplifying a simple declarative sentence in Turkish.

(1) *Bu çocuk bir balon tut-uyor.*this child a   balloon hold-PROG ‘This child is holding a balloon.’

In canonical Turkish sentences with SOV word order as in (1), focus is typically immediately preverbal, provided that no other constituent receives prominent prosodic marking such as stress or pitch accentuation (Erguvanlı, 1984; Göksel and Özsoy, 2000). SOV sentences are also compatible with broad (or sentential) focus, in which the focus domain extends over the entire proposition. That is, the sentence as a whole is informative rather than any single or specific part of it. For example, in (1), the object *bir balon* (“a balloon”) appears immediately before the verb *tutuyor* (“is holding”) and the sentence is therefore compatible with an object focus or broad focus reading. However, appearing in the immediately preverbal position is not the only way to receive focus in Turkish sentences. Other mechanisms, including syntactic movement (Erguvanlı, 1984; İşsever, 2003), prosodic and intonational manipulations such as pitch accentuation (Göksel and Özsoy, 2000), and focus particles (Göksel and Kerslake, 2005; Göksel and Özsoy, 2003; Kamali, 2015; Kamali and Krifka, 2020), can also narrowly focus a part of the sentence. It should be noted that while broad focus presents the whole proposition as informative as in (1), narrow focus restricts the focus domain only to a single constituent.

In this study, we examine the role of one such focus particle, which involves an interesting language-specific device for focus manipulation. In Turkish, focus can be marked by placement of the **question particle (QP) *mI***, which is commonly used to form a Yes-No question sentence, as in (2).

(2) *Bu çocuk bir balon tut-uyor **mu**?*this child a balloon hold-PROG QP ‘Is this child holding a balloon?’

In simple question sentences like (2), the QP *mI*, realized as *mu* by morphophonological rule, commonly appears at the end of the sentence. When the question particle *mu* is attached to the predicate *tutuyor* (“is holding”) in (2), the entirety of the proposition is questioned in Turkish. Of particular interest is that the QP *mu* can also attach to a different constituent in a sentence, and the constituent that it attaches to is a target of focus. For example, if *mu* is attached to the subject noun, e.g., *bu çocuk* (“this child”) in (3) below, it receives focus (Göksel and Kerslake, 2005; Kelepir, 2001).

(3) *Bu çocuk **mu** bir balon tut-uyor?*this child QP a balloon hold-PROG ‘Is it this child that is holding a balloon?’

The interpretation of (3) is equivalent to that expressed by a cleft sentence in English. However, as was discussed above, the foundational syntactic structure in (3) remains like that in (2), unlike, for example, a cleft sentence in English which is argued to be derived via syntactic movement (Chomsky, 1977).

## 3 The acquisition of Turkish focus marking

Turkish is an understudied language in the developmental literature, and existing findings regarding Turkish-speaking children’s focus marking are still sparse. In comprehension, there is one study examining Turkish-speaking children’s comprehension of the focus operator *sadece* (“only”), both in the subject-associated position and in the object-associated position (Topaloğlu and Nakipoğlu, 2017). This study revealed that children are prone to exhibit a subject-object asymmetry, more difficulty comprehending subject-associated *sadece* (“only”), treating it on par with object-associated focus, a pattern also seen languages like English as discussed above. However, no study, to our knowledge, has examined young children’s sensitivity to morphological focus marking via the QP *mI* in comprehension. In what follows, we briefly review the existing evidence regarding children’s production of, and exposure to, the QP *mI*, before turning to the current study which directly examines children’s sensitivity to focus-marking via the QP *mI* in comprehension.

The QP *mI* agrees in person with the noun it is associated with. A longitudinal study by Aksu-Koç and Ketrez (2003) revealed that children nearing 2 years of age start to use the QP *mu* productively and accurately in association with the grammatical person of the noun, despite its morphological complexity. In the adult Turkish morphosyntactic system, the QP *mu* either precedes or follows the person-marker, depending on the agreement paradigm involving person and tense/aspect in verbal morphology, as exemplified in (4–6) for 1st, 2nd and 3rd person singular subjects:

(4)a. *Ben oku-yor mu-yum?* b. *Ben oku-du-m mu?*I read-PROG QP-1^st^SG I read-PAST-1^st^SG QP ‘Am I reading?’ ‘Did I read?’

(5)b. *Sen oku-yor mu-sun?* b. *Sen oku-du-n mu?*you(SG) read-PROG QP-2^sd^SG you(SG) read-PAST-2^nd^SG QP ‘Are you reading?’ ‘Did you read?’

(6)c. *O oku-yor mu?* b. *O oku-du mu?*he/she read-PROG QP he/sheread-PASTQP ‘Is he/she reading?’ ‘Did he/she read?’

For example, if the QP is used with a progressive marker for 1st and 2nd person singular subjects, the person marker follows the QP as in (4a) and (5a), but when it is used with a past tense marker, the person marker precedes the QP as in (4b) and (5b). When the subject is 3^rd^ person singular in a sentence containing a verb with the progressive marker, the person marking is null, as shown in (6); note that this is the configuration that is used in our experimental stimuli, as discussed further below (the subject is always 3^rd^ person singular in our items). Despite the structural complexity in the agreement paradigms in Turkish, longitudinal data indicate that Turkish-speaking children are indeed able to produce the QP in different agreement paradigms by the age of 2, including in the case of a 3^rd^ person singular subject as used in our materials, with the QP appearing with the progressive marker as early as 1;8 (e.g., Aksu-Koç and Ketrez, 2003).

Crucially relevant to the current study, there is also evidence suggesting that Turkish-speaking children can use the QP to indicate the focus of a question in their productions at age 3, which is exactly the role of the QP that we examine in the current study. Recall that the QP can be attached to different constituents in the sentence, and in such cases, the constituent that the QP is attached to becomes focused (Göksel and Kerslake, 2005). The following excerpt from the CHILDES Turkish Aksu Koç Corpus (Slobin, 1982) involves a Turkish-speaking child aged 3 years and 8 months responding to experimenter prompts, attaching the QP to non-predicate constituents to mark focus in a way that conveys a cleft-like interpretation:

(7) EXP: *Niye tırmala-r kedi-ler?*whyscratch-AORcat-PL ‘Why do cats scratch (others)?’ CHI: *Kediler mi?*cat-PL QP ‘(Is it) the cats (that scratch others)? EXP: *Başka hayvanlar-ı gör-dü-n sen sokak-ta?*other animal-ACC see-PAST-2^nd^SG you street-LOC ‘Did you see other animals on the street?’ CHI: *Sokak-ta mı?*street-LOC QP ‘(Is it) on the street (where I saw other animals)?^a^(Child 3;08, File 1fa, Lines 37-41)^a^Morphological glosses and English translations for the utterances were added by the first author.

In this interaction, the child placed the QP on non-predicate constituents in the sentences, as can be seen in the subject in the first CHI tier in (7): *Kediler mi?* “(Is it) the cats (that scratch others)?” and then on the locative adverbial phrase in the second CHI tier in (7): *Sokakta mı?* “(Is it) on the street (where I saw other animals)?” These examples suggest that this Turkish-speaking 3-year-old child is able to use the QP to focalize important information in their productions.

Additionally, evidence from child-directed speech suggests that children are indeed exposed to QP usage in different sentential positions for focus marking. Speech production data discussed in Küntay and Slobin (1996), collected from a mother speaking to her child during the age period of 1;8 to 2;3, provides further evidence for usage of the QP when it is attached to the verb (as in simple Yes/No questions) and instances in which it is attached to other sentence constituents to mark focus in the sentence. The quotes from a mother’s speech in (8) provide examples in which QP attaches to the verb to form a simple Yes/No question (8a), and examples in which the QP attaches to other elements in the sentence to mark focus (8b-c).

(8) a. *Aç-tı-n mı?* (simple Yes/No question) open-PAST-2^nd^SG QP ‘Did you open (it)?’ b. *Kabuk mu ist-iyor-sun?* (QP attaching to the object) peel QP want-PROG-2^nd^SG ‘Is it the peel that you want?’ c. *Bur-da-da mı bubu var?* (QP attaching to the adverb) here-LOC-ADD QP sore exist ‘Is it here that there’s a sore?’

           (Küntay and Slobin, 1996, page: 283)

These patterns provide evidence for Turkish-speaking children’s exposure to relevant input regarding the role of the QP in focus-marking early and robustly (Küntay and Slobin, 1996). The evidence discussed above, taken together, establishes that it is possible that young Turkish-speaking children may indeed possess an adult-like understanding of the role of QP in focus-marking. However, as discussed above, there have been a number of findings in the literature where children appeared to use focus in production but did not use it in comprehension, leading to a debate regarding whether children possess “full competence” that may be masked in comprehension under some circumstances, or “partial competence” reflected by their comprehension difficulties. It is thus crucial to test Turkish-speaking children’s sensitivity to the role of the QP as a focus marker. In this way we address the fundamental issue of to what extent young children may possess adult-like competence regarding focus-marking as realized through morphological marking in Turkish.

## 4 Examining the effect of morphologically-marked attentional focus in Turkish: linking focus marking to universal quantification

The morphological marking of focus via the QP in Turkish, which we examine in the current study, provides an indicator of attentional focus. As is described above, it is a methodological challenge to directly examine the effect of attentional focus marking in comprehension, given that the meaning contribution of this type of focus manipulation is subtle. For example, placing attentional focus on different parts of the sentence does not change its truth-conditional meaning, but rather makes it more or less pragmatically felicitous and potentially more or less easy to process in a given context as discussed below (see, e.g., Szendrői et al., 2018 for further discussion of methodological challenges in testing children’s sensitivity to focus). Thus, what is needed in an experiment like this is not a set of conditions, in which moving focus from one constituent to another would make a sentence true or false according to the adult semantic system, as in traditional applications of child semantic development study designs like truth-value judgment. Instead, a solution may lie in a set of conditions, in which sensitivity to focus-marking could, if the child indeed possesses adult-like knowledge of focus-marking, be used to *facilitate* the comprehension of sentences that are otherwise challenging for young children to process during comprehension. To address this challenge, in the current study we examine children’s sensitivity to morphologically-marked focus in question sentences containing a **universal quantifier** (the Turkish quantifier *her*; which is similar to *every* in English). This type of sentence provides an ideal test case for examining the effect of attentional focus marking in children’s comprehension for two reasons, each of which we discuss in detail below: (i) sentences with a universal quantifier have been clearly shown, across languages, to be challenging for young children to comprehend; (ii) recent findings in this literature suggest that children’s difficulties in comprehending quantified sentences do not appear to be due to a lack of adult-like competence regarding quantification, but instead to challenges in using this knowledge during comprehension. Importantly, several factors have been proposed to resolve children’s challenges in using the knowledge, including factors that help children attend to things in the context that are relevant to interpreting the quantified sentence, rather than to salient but irrelevant things. Given that focus manipulation serves to cue what is relevant (and hence focalized) in the given discourse are, this crucially yields a test case. There are known challenges for young children in comprehension, but they can be ameliorated if they receive helpful support for focusing their attention. This is exactly what we have argued above that the Turkish QP *mI* can provide, but only if children indeed possess the relevant knowledge of the Turkish QP *mI* as an attentional focus marker.

As regards children’s comprehension of sentences containing a universal quantifier, there has been a body of literature since at least as far back as Piaget (1951), which suggests that children are prone to exhibit patterns of erroneous comprehension of this type of sentence. The typical error that children make is often referred to as **quantifier spreading** (**q-spreading**). To illustrate, one typical form of q-spreading error is shown as erroneous rejection of a statement with a universal quantifier, e.g., *every* in English associated with the subject, particularly when the statement is actually true but presented along with a specific type of error-inviting depictions, known as an “**extra object”** pictures. For example, suppose that a participant is asked, “*Is every child is holding a balloon*?”, and presented an extra-object picture which depicts some children holding a balloon each and an extra balloon held by nobody (as in Figure 1 below). Logically, the correct answer to this question is “Yes”, because the picture matches the proposition “Every child is holding a balloon.” even though there is an extra balloon no child is holding. When the participant erroneously answers “No” and points to the extra balloon as their justification of their negative response, they exhibit a q-spreading error. The response and its justification are not target-like, because what is relevant for the adult-like interpretation of the sentence is whether each child holds a balloon (which was true in the picture) and not whether there are other balloons that are not held by anybody (which there was, but that is not relevant for interpreting the sentence and answering “Yes” or “No”).

**Figure 1:**
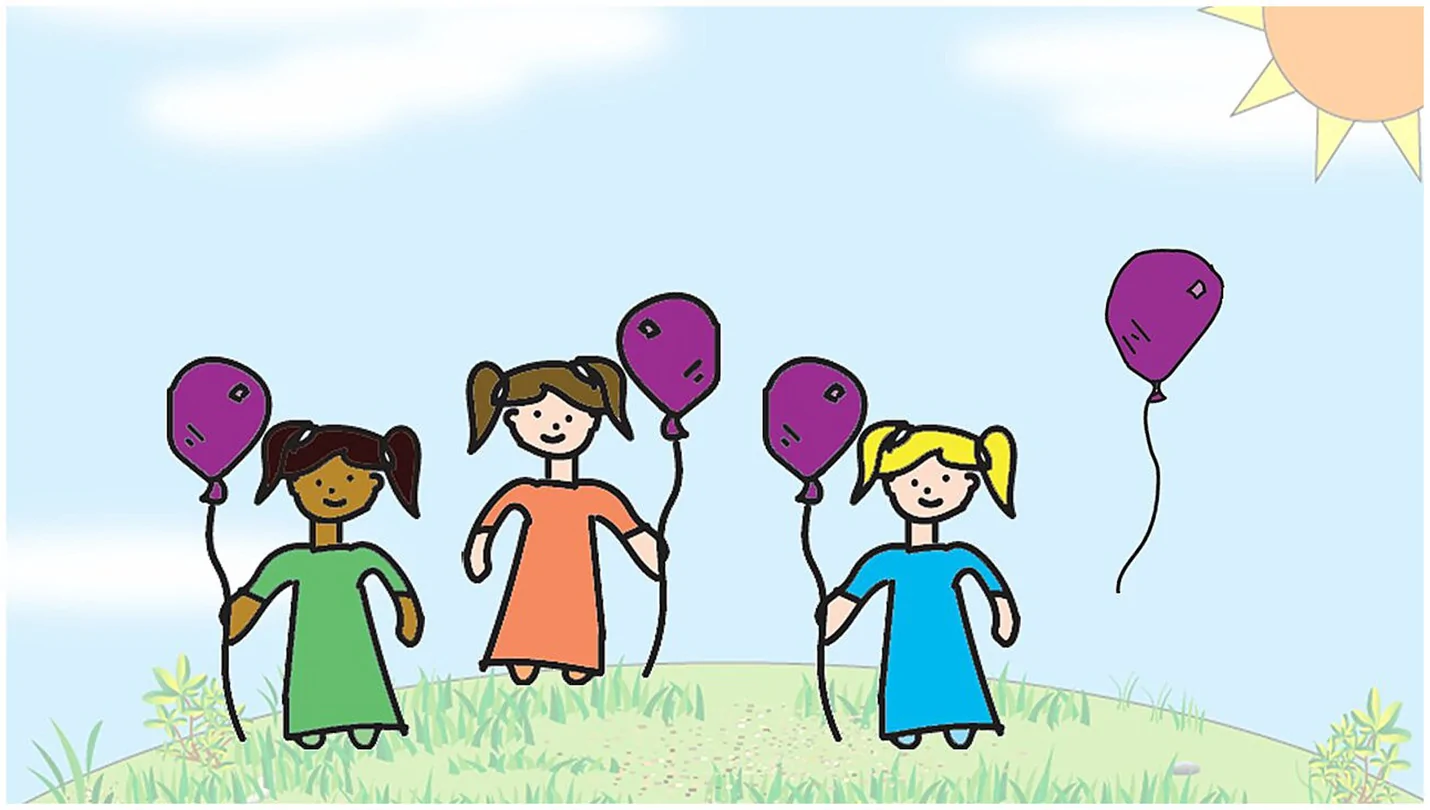
Example of an extra-object picture paired with the test sentences (9–10).

A major debate has arisen regarding the origin of children’s q-spreading, generating two alternating camps of accounts, a “partial competence view” and a “full competence view”, paralleling the debate in the focus literature regarding to what extent children possess adult-like competence in that domain, as discussed above. Under the “partial competence” view, q-spreading is accounted for by a lack of adult-like linguistic knowledge in the child system which is required to implement correct semantic representation of a universally-quantified sentence (e.g., Drozd, 2001; Philip, 1995). Under the “full competence” view, q-spreading is attributed to extralinguistic factors, including discourse factors (e.g., Crain et al., 1996; Gouro et al., 2001; Kiss and Zétényi, 2017; Skordos et al., 2022; Sugisaki and Isobe, 2001), cognitive factors (e.g., Aravind et al., 2017; Minai et al., 2012) and task-related factors (O'Grady et al., 2010).

One factor we focus on here is the role of attention. This factor has been proposed under the Full competence view to play an important role in accounting for q-spreading errors. In particular, the extra object (e.g., the balloon that is not held by anybody, in Figure 1) may be particularly attention-drawing, despite being irrelevant to the semantic evaluation of the meaning of the universally-quantified sentence. As such, some researchers have claimed that the **visual salience of the extra object** in the picture may be a contributing factor to children’s q-spreading. For example, Sugisaki and Isobe (2001) analyzed the design of the extra object pictures like Figure 1 and hypothesized that the extra object, uniquely unpaired with any agent, stands out as an outlier, thus becoming visually salient. To test this, they utilized a modified version of the extra object picture layout. In their modified version, the number of “extra” objects not paired with the agent outnumbered the agent-object pairs. (In our balloon example, that would mean there would be more balloons that are not held than balloons that are held). They found that the modified extra object picture design elicited significantly less q-spreading from children (also see Gouro et al., 2001 for the effect of attenuated visual salience achieved via a different modification to the extra object pictures). Sugisaki and Isobe (2001) argue that, in their modified extra object pictures, it was the object-agent pairs that served as “outliers” receiving visual salience, hence, as the “figure” in the visual scene, while the outnumbering agent-less “extra” objects, by contrast, served as the “ground”. Hence, the extra objects no longer drew undue attention, allowing children to avoid q-spreading.

Elaborating this account, Minai et al. (2012) adopted the Attentional Inertia hypothesis (e.g., Diamond et al., 2005) and hypothesized that children’s q-spreading is a result of their failure in shifting their attention from the visually salient extra object and reengaging to evaluate the picture with respect to the quantification of the characters, due to their still-developing ability to shift perspectives. For example, when viewing an extra-object picture depicting children holding a balloon each and an extra balloon nobody is carrying, children initially pay attention to the presence of the visually salient extra balloon. When hearing the sentence *Is every child holding a balloon?*, the presence of the extra balloon is irrelevant for the current task of deciding whether to answer *Yes* or *No*, because the presence/absence of the extra balloon neither verifies nor falsifies the proposition about every child’s carrying a balloon. However, once children perceive the picture as a depiction of the salient extra balloon, they are no longer able to disengage from that perspective. Even though their task is now to answer the Yes/No question, for which the presence of the extra balloon no longer plays any role, they are unable to establish a new perspective. As such, they cannot reinterpret the picture as a depiction of children carrying balloons and evaluate it with respect to whether or not all the children are carrying a balloon. To test this hypothesis, Minai et al. (2012) conducted a picture-sentence verification experiment, during which children’s eye movements were measured via eye-tracking. Children also completed the Dimension Change Card Sort (DCCS: Zelazo, 2006) to examine children’s mental flexibility required for perspective shifting. The results revealed that: (i) children dichotomously split into an adult-like cluster, seldom showing q-spreading, and a spreader cluster, robustly showing q-spreading; (ii) those in the spreading cluster showed more robust eye looks to the extra object than those in the adult-like cluster, and; (iii) those in the spreading cluster performed more poorly in the DCCS than those in the adult-like cluster. Based on these findings, Minai et al. (2012) claim that children’s q-spreading may stem in part from their still-developing cognitive abilities, hindering successful perspective shift. As such, they were unable to disengage from attending to the salient but irrelevant information (extra object) and reengage from a new perspective. Taken together, they concluded that children’s over-engagement with the extra object and their failure to disengage from it due to their still-developing mental flexibility led them to commit q-spreading.

Note that, in the studies discussed above, visual salience was argued to draw robust attention from participants; recall that this is also the function that we ascribed to attentional focus marking. Here we identify a parallel between visual salience manipulation and attentional focus manipulation, both of which share the property of directing attention. This parallel allows us to link the quantification literature to the focus literature and examine whether attentional focus marking may facilitate children’s quantifier comprehension, as manipulation of visual salience did in the studies discussed above. Specifically, we examine whether morphological focus marking in Turkish, and more specifically focusing a universally-quantified subject with the QP *mI*, may help facilitate children’s comprehension of such sentences when presented with a typical extra object picture, causing them to show fewer q-spreading errors. Finding such a pattern would provide evidence that children can indeed demonstrate adult-like knowledge of morphologically-marked focus in comprehension, as would be expected under the “full competence” view of focus, while also yielding new evidence to the increasing body of literature supporting the “full competence” view of quantification as well.

We thus address the following research question in the current study: Does focusing a quantified subject with a QP serve as a cue for Turkish-speaking children to correctly comprehend universally-quantified sentences, helping them avoid q-spreading? The current study is the first to examine child Turkish, in terms of whether children can incorporate QP placement as a marker of attentional focus during sentence comprehension. We also newly investigate whether Turkish-speaking children also exhibit q-spreading when comprehending sentences with the universal quantifier, as was attested for children speaking a range of languages, including English (Aravind et al., 2017; Crain et al., 1996; Minai et al., 2025; Philip, 1995; Skordos et al., 2022), Japanese (Gouro et al., 2001; Minai et al., 2012; O'Grady et al., 2010; Sugisaki and Isobe, 2001), Korean (Kang, 2001), Russian (Sekerina and Sauermann, 2017), Hungarian (Kiss and Zétényi, 2017), and Spanish (Escobar and Torrensm, 2008). Lastly, we newly investigate whether their q-spreading can be ameliorated by factors outside of quantification, like attention, as would be expected if “full competence” in the domain of quantification holds in child Turkish.

## 5 Experiment

### 5.1 Participants

We tested 18 monolingual Turkish-speaking children (age range = 4;0–5;10, mean age = 4;11). All child participants were living in Turkey at the time of testing and were attending to a kindergarten or pre-school in which the medium of instruction was primarily Turkish. According to parental reports on a language background questionnaire, the children were predominantly monolingual Turkish speakers, with only minimal second-language (English) exposure through schooling. None of the children were reported to have a diagnosed language or developmental disorder. We also tested a control group consisting of 18 adult native speakers of Turkish (age range = 18–38, mean age = 29.17) who were also residing in Turkey at the time of testing.

### 5.2 Design and materials

The experiment was designed with the structure of target stimulus sentences (Yes-No questions containing an *every*-quantified subject) as the primary independent within-subject factor. We developed two types of Yes/No question sentences in Turkish, with a universally-quantified subject, a simple transitive verb and an object noun phrase, to serve as test sentences in our two experimental conditions. The primary experimental condition was the **Focused *every* Condition**, in which the test sentences were Yes/No question sentences that the QP (*mI*) attached to the universally-quantified subject noun phrase, placing attentional focus on the subject (9).

(9) *Her çocuk **mu** bir balon tut-uyor?*every child QP a balloon hold-PROG ‘Is it every child that is holding a balloon?’

In (9), the QP *mu* marks the universally-quantified subject noun phrase *her çocuk* (“every child”) as the target of the focus, conveying a cleft-like narrow focus interpretation, as is presented in the translation above. This condition allows us to test if focus marking on the quantified subject may help Turkish children correctly interpret universally-quantified sentences in an extra-object context by drawing attention to the quantified subject. Adopting Skordos et al. (2022), a recent study examining English-speaking children’s comprehension of sentence with the universal quantifier, testing for q-spreading errors using a picture-sentence verification task, we developed a total of five test sentences for the Focused *every* Condition. The other condition is the **Baseline Condition,** which consisted of simple Yes/No questions, as in (10).

(10) *Her çocuk bir balon tut-uyor **mu**?*every child a balloon hold-PROG QP ‘Is every child holding a balloon?’

In the Baseline Condition sentences, the QP *mu* is attached to the predicate *tutuyor* (“is holding”) as in (10), so the question targets the entire proposition rather than a certain constituent (such as the universally-quantified subject) in the sentence. The five target sentences for the Baseline Condition constituted minimal pairs with their corresponding Focused *every* Condition sentences, differing only with respect to the position of the QP. As the translation in (10) shows, these sentences correspond to simple Yes/No questions with a neutral focal interpretation. The Baseline Condition sentences are similar to those tested in a wide range of other languages that were found to robustly elicit children’s q-spreading in the existing literature. This condition thus demonstrates whether q-spreading occurs in Turkish. Additionally, comparing the Baseline and the Focused *every* conditions will demonstrate whether sensitivity to the focus marker facilitates comprehension of the test sentences, reducing the occurrence of q-spreading in the Focused *every* Condition compared to the Baseline Condition.

As visual stimuli, we created a set of five extra-object condition picture materials to be paired with each of the target sentences in both the Focused *every* Condition and Baseline Condition. These pictures followed the typical extra object picture layout; there was one extra object that was not being acted on by any agent in the picture. Figure 1 provides an example extra-object condition picture corresponding to the test sentences in (9–10).

Developing these two conditions allowed us to examine the effect of attentional focus manipulation as follows. In the Focused *every* Condition, as in (9), the universally-quantified subject noun *her çocuk* (“every child”) is marked with the QP *mu*, which conveys the cleft-like, narrow focus interpretation. In the Baseline Condition, on the other hand, a broad focus reading obtains (10). If the attentional focus on the universally-quantified subject was effective, and the participants’ attention was allocated to the subject, it may have helped them disengage from paying attention to the visually salient extra object and avoid showing q-spreading, i.e., erroneous “No” responses justified by the presence of the extra object in the picture. Such a scenario would be borne out if results revealed lower q-spreading rates, i.e., higher rates of the adult-like/accurate “Yes” responses for the Focused *every* Condition than the Baseline Condition.

Following Skordos et al. (2022), we also created filler items: five additional Yes/No question sentences with a universally-quantified subject, none of which were presented with extra-object pictures. Similar to the approach used for target items, the QP followed the universally-quantified subject in filler items in the Focused *every* Condition. A sample filler sentence in the Focused *every* Condition is given in (11), and a sample in the Baseline Condition in (12). To counterbalance, two of the filler items were paired with a picture that matches (and verifies the truth of) the sentence. For those fillers, what the question sentence asked about was true in the picture and thus targeted “Yes” answers. The remaining three fillers are pictures mismatching (and falsifying) the sentence, where the question asked about what was not true in the picture and thus targeted “No” answers. Unlike the target items with “extra object” pictures eliciting “No” responses as a result of q-spreading, these “No”-eliciting fillers were presented with logically mismatching pictures. Figure 2 provides a sample picture for the filler sentence (11) used in the Focused *every* Condition and (12) used in the Baseline Condition. Note that, in Figure 2, the presence of an extra cat that is not sleeping logically falsifies the proposition (Every cat is sleeping) in (11–12).

(11) *Her kedi mi uyu-yor?*every cat QP sleep-PROG ‘Is it every cat that is sleeping?’

(12) *Her kedi uyu-yor mu?*every cat sleep-PROG QP ‘Is every cat sleeping?’

**Figure 2:**
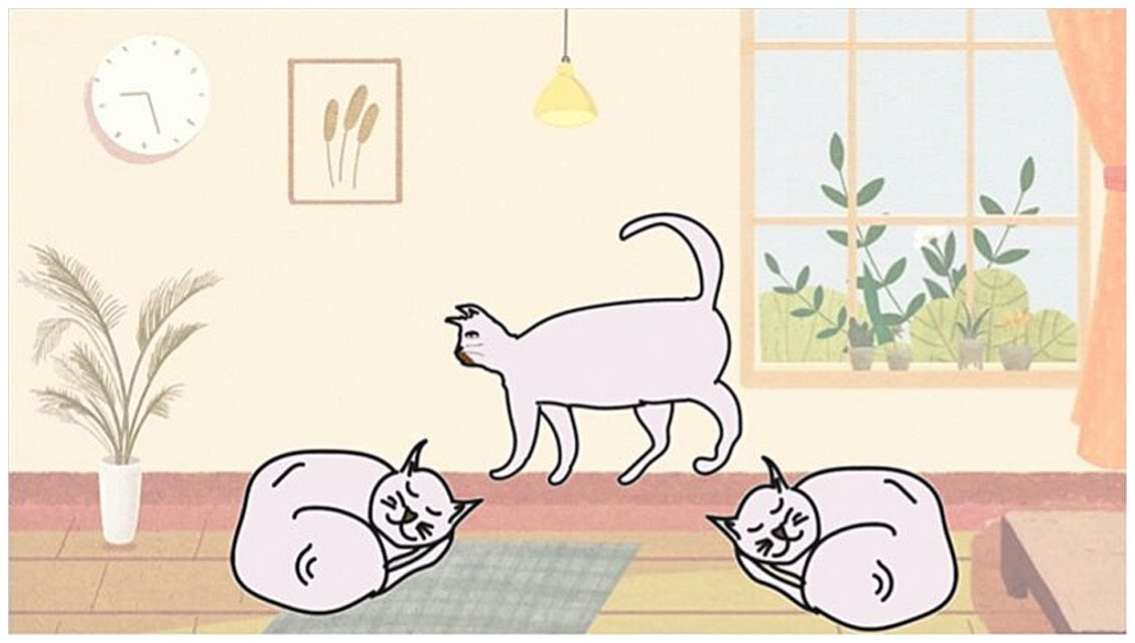
Example of a filler item picture.

All the stimulus sentences were pre-recorded and created as audio files. A female native speaker of Turkish audio-recorded each of the test sentences with a natural, child-friendly prosody. A list of all the test sentences is provided in Appendix A.

We alternated target and filler items and created two order lists: one order, and its reverse. Each child was assigned to see the items in one order for the Baseline Condition, and see them in reverse for the Focused *every* Condition.

### 5.3 Task and procedures

The main experimental task was a picture-sentence verification task designed similarly to the one in Skordos et al. (2022), in which participants viewed pictures and answered Yes/No questions. For each condition, there were 5 target items, in which there are cartoon characters engaging in an activity and an extra object (presented as “extra object” pictures), as well as 5 filler items. There were 2 practice items at the start of each condition. On each target trial, participants first saw an extra-object picture and heard a Yes/No question with the universally quantified subject in Turkish (9–10). They were then asked to answer the question orally. Their responses to the Yes/No question indicate how they evaluated the match (truth) or mismatch (falsity) between the depiction of the picture and the sentence’s meaning. If they thought that the picture and the sentence match (true), they were asked to say *Evet* (“Yes”), but if they thought that the picture and the question did not match (false), they were asked to say *Hayır* (“No”). After this step, they were also asked to provide justification for their responses (for both accepting and rejecting a given statement), by answering the additional question “Why did you think so?”. Collecting the participants’ justifications for their responses allowed us to decide whether “No” response to the target items indeed reflected a q-spreading error (i.e., if q-spreading, their justification for “No” response should be attributed to the presence of the extra object in the picture). Their answers were manually recorded on a score sheet.

Testing of child participants was administered in two Zoom video conferencing sessions scheduled on different days, involving the child, the parent, and the experimenter. Prior to testing, the parent was instructed not to interact with the child during testing. The testing of children took approximately 5 to 10 minutes, and the entire testing session was recorded via Zoom. We used a within-subject design in which each child participant was tested twice on different days, once with the Baseline Condition and once with the Focused *every* Condition. All the participants took the Baseline Condition in their first session, and then completed the Focused *every* Condition in their second session. This allowed us to obtain baseline child data unaffected by previous exposure to the critical experimental contexts. The two sessions were spaced at least 1 week apart to minimize carryover effects.

In the first session, the parents were asked to give their consent for participation in the two-session experiment and complete a questionnaire form gathering information about their child’s age, gender and language information, as well as the parents’ educational background. This part took approximately 15 min to complete. Then, the child was asked to give their assent, and then the actual experiment began, as described above. The second session followed the same procedure, except that parental consent and the background questionnaire were not repeated. The first session took approximately 25 minutes, and the second session took around 10 minutes.

Testing of adult participants took place on the Qualtrics survey platform; the audio files for the test sentences and the visual files for the pictures was combined in slides for viewing via the internet. We used a between-subjects design for adult participants in that they were tested only once and assigned to one of the two experimental conditions, as the goal was to establish that the stimuli elicited the expected adult-like responses, rather than to test for the effect of focus on q-spreading. The experimental task and procedures were the same for the adult controls in that they were required to hear a pre-recorded sentence and view a picture, and judge whether there was a match or mismatch between the picture they saw and the sentence they listened to. The sentence stimuli and visual stimuli used in the experiment for adults were the same as the materials used in the children’s experiment. The completion of the entire task took approximately 10 minutes in total.

## 6 Results

Children’s responses to each question were coded as adult-like when the child returned a logically correct answer (1) or as an error if not (0). All analyses were conducted on these trial-level binary responses (18 children × 5 items × 2 conditions = 180 observations). Condition was manipulated within participants, and each of the five target items appeared in both conditions.

Figure 3 summarizes children’s performance as the proportion of adult-like responses per child in each condition. Note that the q-spreading responses were errors and hence coded as 0. This means that higher rates of accurate, adult-like responses indicate lower rates of q-spreading errors. Children were more accurate in the Focused *every* Condition (*M* = 0.68) than in the Baseline Condition (*M* = 0.58). Adult participants performed at high accuracy across both experimental conditions (Baseline = 0.93; Focused *every* = 1).

**Figure 3:**
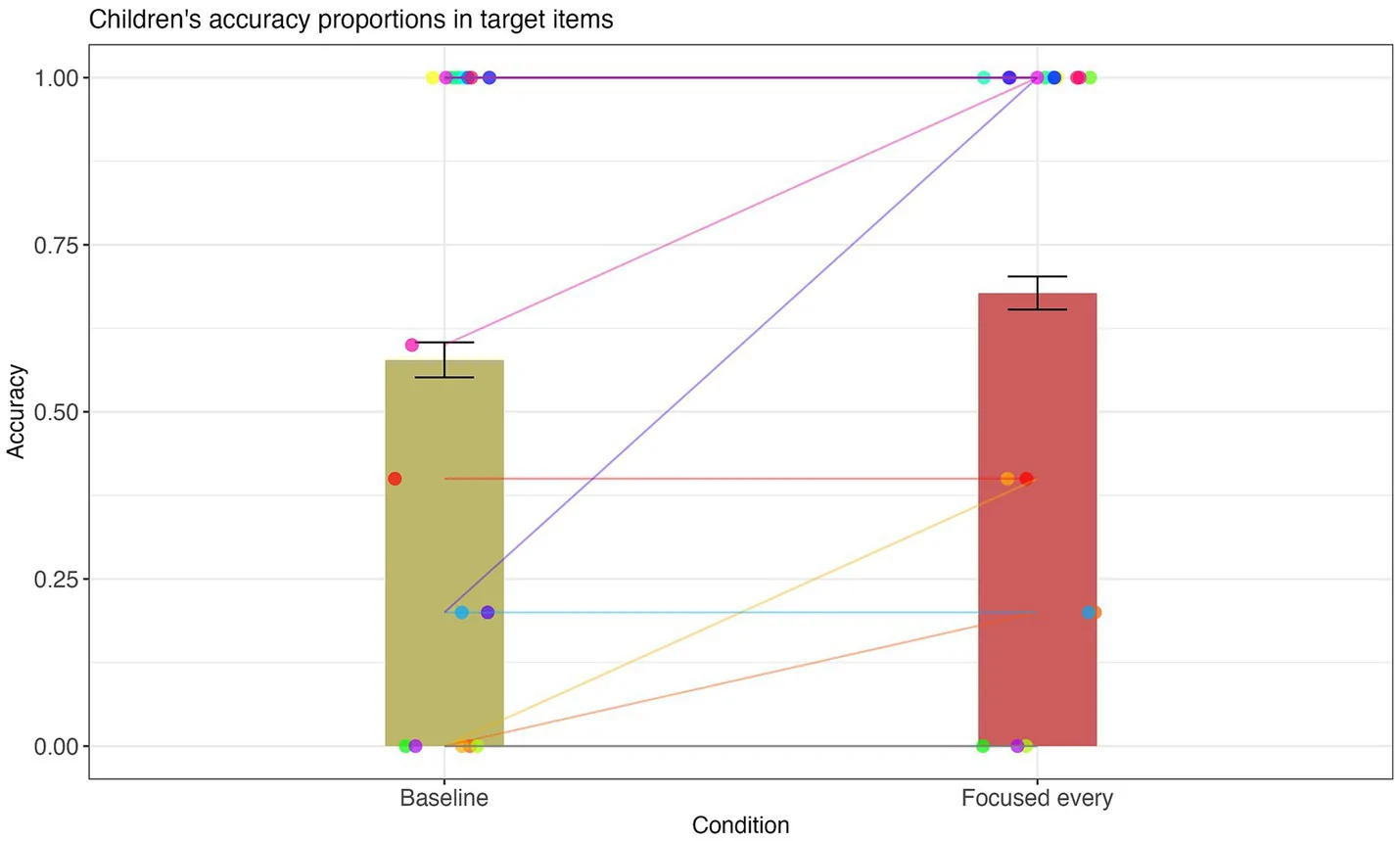
Children’s mean proportion of correct responses for each condition. Scatterplots represent each child participant’s accuracy for each condition, connected with a trendline across conditions.

Following Skordos et al. (2022), we first fitted a mixed-effects logistic regression using the *glmer* function from the *lme4* package (Bates et al., 2015) in R (R Core Team, 2024), with Condition as a fixed effect (dummy coded, Baseline Condition as reference level) and by-subject and by-item random intercepts. A model including Condition was compared against a model containing only the intercept and the same random-effects structure^1^ via a likelihood ratio test, which indicated a significant effect of Condition, *χ*^2^(1) = 8.73, *p* = 0.003. However, its estimates were unstable. The reason is that most children provided responses uniformly across both conditions, such that nine children were at ceiling (5/5 correct) in both conditions and three children were at floor (0/5) in both, leaving six children whose responses varied in the Baseline and Focused *every* conditions. This pattern, known as quasi-complete separation, is a well-documented source of instability in mixed-effects logistic regression, and its consequences were evident in our initial analyses. More specifically, the model returned an implausible by-subject standard deviation of 13.25 on the log-odds scale and predicted probabilities above 0.999 in both conditions, even though we observed accuracy of 0.58 for the Baseline condition and 0.68 for the Focused *every* condition.

We therefore turn to a Bayesian model, using the *brms* package (Bürkner, 2017) in R, in which weakly informative priors yield stable estimates under these conditions. Condition was again included as a fixed effect, and Subject and Item were included as random intercepts into the model. We used dummy coding similar to our initial analysis, with Baseline Condition as reference level. This model converged without difficulty, and the by-subject standard deviation was reduced to 6.55, compared with 13.25 under maximum likelihood.

The Bayesian model also confirmed the effect of Condition; children were more likely to give adult-like responses in the Focused *every* Condition than in the Baseline Condition, *β* = 1.74, 95% CrI [0.44, 3.13]. Expressed as probabilities, the model estimated that an average child would respond in an adult-like way on 0.89 of trials in the Focused *every* Condition, compared with 0.68 in the Baseline Condition, yielding a difference of 0.21. The posterior probability that this difference was positive was 0.997, suggesting that the Bayesian model places almost all of its belief on children performing better in the Focused *every* Condition.

We then performed Bayes factor analyses to quantify the relative evidence for the null and alternative hypotheses. Following Dienes (2014), when Bayes factors are reported as BF10, values greater than 3 indicate substantial evidence for a difference between conditions, whereas values less than 0.33 indicate substantial evidence for the absence of a difference. A Bayes factor comparing the full model with Condition to an equivalent model without Condition, estimated via bridge sampling (Gronau et al., 2020), provided strong evidence for the effect of Condition, BF10 = 13.71, demonstrating that the observed data were approximately 13.7 times more likely under the model including Condition than under the model without it. Together with the direction of the estimated probabilities, the Bayes factor analysis provides further support for children’s higher accuracy in the Focused *every* Condition than in the Baseline Condition in the present study.

To examine whether there was individual variation in response patterns across two conditions from child to child, we also investigated each child’s response patterns across the two conditions. The scatterplots in Figure 3 present individual child participants’ mean accuracy for each condition, and a line connects the datapoints from each child across conditions, individually color-coded for each child. This additional analysis revealed individual differences among children with respect to how they responded in each condition. Of all the 18 children, 12 children either consistently committed q-spreading or were consistently adult-like; these individual differences are consistent with patterns observed in previous studies in this literature (e.g., Minai et al., 2012). Within this group of 12 children, 3 children consistently provided q-spreading errors across both conditions, without providing any correct *Yes* answers across both the Baseline Condition and the Focused *every* Condition. Nine children, on the other hand, consistently showed adult-like performance, providing *Yes* responses to all the items in both the Baseline Condition and the Focused *every* Condition. Among the remaining 6 children, 2 children returned mixed responses with a chance-level mean across conditions. Crucially, the other 4 children showed more adult-like *Yes* responses for the Focused *every* Condition compared to the Baseline Condition, and no children showed better performance in the Baseline Condition compared to the Focused *every* Condition. The findings reveal more adult-like responses for the Focused *every* Condition than for the Baseline Condition, but not vice versa. Results therefore suggest that our experimental manipulation of focus marking on the universally-quantified subject noun phrase may indeed help children avoid committing q-spreading errors.

It should be noted that the 12 children who responded uniformly across both conditions could not, by definition, contribute information about the effect of Condition. The estimate is therefore driven by the six children whose responses varied in the Baseline and Focused every conditions. Among these, four children responded more accurately in the Focused *every* Condition, and none in the Baseline Condition.

We also examined whether variability among children was related to their age. In a Bayesian model including mean-centered Age and its interaction with Condition, the effect of Condition remained credible, *β* = 2.01, 95% CrI [0.66, 3.55], but neither the main effect of Age, *β* = −0.35, 95% CrI [−3.66, 3.04], nor its interaction with Condition, *β* = 1.35, 95% CrI [−0.84, 3.69] was estimated with sufficient precision to support a conclusion. We argue that with 18 children with a relatively narrow age range in the present study, the design was not powerful enough to detect variability potentially influenced by children’s age. We therefore treat these estimates as inconclusive rather than as evidence for the absence of an age effect.

Children also performed almost at ceiling for filler items such as (11–12) in both the Baseline Condition (accuracy proportion = 0.94) and the Focused *every* Condition (accuracy proportion = 0.99), suggesting that they had a clear understanding of the task and were able to accurately interpret the filler items with a universal quantifier when presented with a picture different from the extra object condition. This consistently high level of performance on filler items across conditions also indicates that the differences observed between the experimental conditions are likely due to the stimulus manipulations rather than a general inability to comprehend the universal quantifier in Turkish.

### 6.1 Summary of findings

The results suggest that QP focus marking on the quantified subject noun phrase can facilitate Turkish-speaking children’s comprehension of universal quantification, reducing the rate of q-spreading errors. When the QP marks focus on the universally quantified subject in an extra-object setting as in the Focused *every* Condition (9), children overall provided more adult-like responses compared to the Baseline Condition with simple Yes/No questions (10).

Our findings also revealed that a number of the children’s split into two clusters based on their response patterns across conditions, either showing consistent adult-like responses or showing consistent q-spreading, replicating previous findings (Minai et al., 2012, 2025). Note that, whereas children from previous studies who showed this split were tested with a single condition with the extra-object condition, children in the current study, adopting a within-subject design, were tested twice, both with the Baseline Condition (replicating the traditional extra-object condition known to elicit q-spreading) and with the experimental, Focused *every* Condition. Findings revealing children’s split across both conditions suggested that some preschool-age children are prone to commit q-spreading while others are not, even when the experimental discourse was manipulated within a single study. We also found that, whereas a cohort of children showed a consistent response pattern across conditions, either presenting adult-like responses or showing q-spreading errors to all the items for both conditions, there was a group of children who could return more adult-like responses for the Focused *every* Condition than for the Baseline Condition. The findings of the last group of children clearly show effects of focus marking at the individual level, which facilitated their comprehension of quantified sentences, leading to a reduced rate of q-spreading.

Taken together, these findings suggest that Turkish children are sensitive to morphologically-marked focus instantiated via the QP, utilizing QP-marked focus as a cue to attend to the universally-quantified subject noun phrase. This facilitated their interpretation of the question as one about the subject referents, e.g., whether all children were balloon-holders, rather than one about an extra object, e.g., an extra balloon nobody was holding. This effect of attentional focus in reducing q-spreading errors is consistent with previous findings that manipulated attention in other ways, such as visual salience (Gouro et al., 2001; Minai et al., 2012, 2025; Sugisaki and Isobe, 2001).

## 7 Discussion

### 7.1 Comprehending attentional focus

The present study investigated the role of attentional focus marking and its effect on sentence comprehension in preschool-age children. Specifically, we examined Turkish-speaking children’s comprehension of focus manipulation via QP placement. Existing child Turkish data only suggested their early use of QP focus marking in production (Aksu-Koç and Ketrez, 2003; Slobin, 1982) and exposure in the adult input (Küntay and Slobin, 1996). The current findings are the first to suggest Turkish-speaking children’s early awareness of the contribution of attentional focus marked by QP placement, providing evidence of preschool children’s incorporation of focus manipulation into sentence meaning comprehension.

Note that previous studies on the acquisition of focus, across a range of languages, have revealed mixed results in terms of preschool children’s awareness of focus marking in their comprehension, both as regards focus marking through a focus particle (e.g., *only* in English, Crain et al., 1994; Kim, 2011; Notley et al., 2009; Paterson et al., 2003) and as regards prosodic focus marking (e.g., Sekerina and Trueswell, 2012; Wells et al., 2004). Previous work also generated alternating accounts: those arguing that difficulties in comprehension reflect “partial competence” (e.g., Cutler and Swinney, 1987) and those arguing that those difficulties are due to factors outside of the knowledge of focus, consistent with claims of “full competence” in the domain of focus (e.g., Höhle et al., 2009a; Szendrői et al., 2018). The current study specifically examined a unique, language-specific method to manipulate attentional focus in Turkish, morphological focus marking without the syntactic complexity of clefting. Our findings revealed that children’s comprehension of quantified sentences (known to robustly elicit q-spreading errors) could be improved, when subject was attentionally focused. These findings suggest that children are able to incorporate morphologically-marked attentional focus into their comprehension when unimpeded by syntactic complexity. The current study examined a linguistic instantiation of focus that has not yet been discussed in the literature. Our findings support the “full competence” claim that children do not lack the competence for manipulating linguistic focus (e.g., Höhle et al., 2009a; Szendrői et al., 2018), suggesting that children’s improved quantifier comprehension may be attributable to the effect of focus manipulation.

It should be noted, however, that the current study did not include a condition in which children’s comprehension of focus marking was directly measured alone. Although this would not preclude the interpretation above, we would like to note that it warrants some caution in drawing a strong conclusion in favor of the “full competence” claim from our findings^2^. As was discussed above, the literature revealed that, when a contrastive focus particle (e.g., *only* in English) is placed on the subject, children are known to erroneously associate it with the object (e.g., Crain et al., 1994; Kim, 2011; Notley et al., 2009; Paterson et al., 2003; Endo, 2004; Höhle et al., 2009a; Szendrői et al., 2018). In the current study, we focus-marked the universally-quantified subject, creating a discourse where children tend to wrongly associate the subject-modifying focus with the object. We did not utilize a subject-modifying contrastive focus particle in Turkish *sadece* (“only”), which Turkish children are known to incorrectly associate with the object (Topaloğlu and Nakipoğlu, 2017). We do not know whether QP-marked attentional focus shows the same effect. It is an open question whether, and to what extent, children in our study correctly associated the focus with the subject, given that we did not include a direct measure.

In light of this, we address the analogy between contrastive focus computation and universal quantification and present another way to interpret the current findings. In children’s comprehension, both subject-modifying contrastive focus particles and universal quantifiers tend to be incorrectly interpreted in association with the object. In such a sense, the incorrect association of subject-modifying focus with the object resembles q-spreading. When “Every child is carrying a box” is interpreted wrongly by children who show q-spreading, they reject the sentence based on the presence of the extra box, even though its presence has no basis for evaluating the meaning of subject-modifying *every*. When “Only a child is carrying a box” is interpreted wrongly by children associating “only” with the object, they incorrectly search for anything *in addition to the box* that the child is carrying. Given this, when focus is erroneously comprehended in association with the object, it may serve as an extra cue to draw attention to the presence of the (extra) object, in addition to the presence of the extra object in the picture. If both cues cumulatively bias children towards attending to the (extra) object(s) in the scene, it may have been reflected as increased rates of q-spreading for the Focused *every* Condition (where both the extra-object pictures and the subject-marking focus were present) as compared to the Baseline Condition. However, our results revealed the opposite effect, higher rates of q-spreading for the Baseline Condition than for the Focused *every* Condition. Hence, our findings still suggest that correct interpretation of subject focus attenuates q-spreading, aligning with the “full competence” claim.

### 7.2 Attentional focus cuing quantification

We examined morphological focus marking in Turkish, where focus marking QP (*mI*) places attentional focus on a universally-quantified subject without added syntactic complexity. Our hypothesis was that the linguistic manipulation of attentional focus given in the Focused *every* Condition may work similarly to manipulations of visual salience in the extra object (e.g., Minai et al., 2012; Sugisaki and Isobe, 2001), helping children avoid paying excess attention to the extra object and generating fewer q-spreading errors, compared to when the focus was on the entire preposition as in the Baseline Condition. We found that children have lower rates of q-spreading errors for the Focused *every* Condition than in the Baseline Condition, suggesting that Turkish-speaking children are able to incorporate QP-marked attentional focus and enhance their comprehension of universal quantifier. These findings suggest that children are sensitive to the morphological manipulation of attentional focus in comprehension, in support of the “full competence” view of focus. The within-subject design we adopted further allowed us to find individual differences. While some children consistently committed q-spreading and others were consistently adult-like across conditions (replicating previous findings with children acquiring other languages), all the children who changed the response patterns showed *improved* quantifier interpretation with QP-marked subject focus, with lower rates of q-spreading for the Focused *every* Condition than for the Baseline Condition, and not vice versa.

Crucially, the current study demonstrated that attentive focus manipulation, even though its contribution to the sentence meaning is subtle, can be effectively assessed with young children in domains like universal quantification, where focus-marking facilitates adult-like comprehension. Our findings are consistent with those of previous studies supporting the “full competence” view in the universal quantifier literature, which attribute children’s q-spreading to performance factors like the visual salience in the extra object that attracts excess attention (Gouro et al., 2001; Sugisaki and Isobe, 2001) and children’s challenges in suppressing attention-drawing but irrelevant information due to their still-developing executive function (Minai et al., 2012). Our results suggest that QP-marked subject focus might have helped children disengage from paying too much attention to the extra object (despite its attention-drawing nature), and engage in evaluating a question about the subject. Hence, the current study demonstrated that attentional focus marking may help children avoid committing q-spreading errors, in a similar way to other performance factors proposed under the “full competence” camp, such as pragmatic factors that improve felicity/plausibility of quantification (e.g., Crain et al., 1996), visual factors inherent to the extra-object condition (Gouro et al., 2001; Sugisaki and Isobe, 2001) or cognitive factors (Minai et al., 2012). Our findings serve as the first in the universal quantifier literature to suggest that a language-specific factor created by grammatical manipulation of test sentences, such as morphological focus marking, may also modify an experimental discourse in a way to elicit more adult-like comprehension responses from children. Our findings provided new evidence consistent with accounts in which children are better able to arrive at adult-like interpretations of sentences containing the universal quantifier when their attention to relevant information is facilitated, rather than being limited in their ability to comprehend quantified sentences due to possessing qualitatively non-adultlike semantic knowledge (e.g., Philip, 1995).

Whereas we underscore the importance of our findings suggesting an effect of language-specific factors helping children avoid q-spreading, there are considerations that should be kept in mind when interpreting our findings. Although we found a significant difference in rates of adult-like responses (as indication of q-spreading avoidance) between conditions, the rates of adult-like responses in the Focused *every* Condition (0.68) is below 0.7 and lower than some previous findings in the literature that reported increased rates of adult-like responses in the target/experimental condition (e.g., 0.78 in Skordos et al., 2022; 0.875 in Sugisaki and Isobe, 2001; 0.718 in Gouro et al., 2001; 0.88 in Crain et al., 1996).^3^ While we acknowledge that it is an issue to be addressed in future research, we note that some methodological factors, such as within-subject design (which has seldom been adapted for studies examining q-spreading), the remote testing format and the limited sample size, may have contributed to suppress the accuracy rates in the current study.

### 7.3 Limitations and future directions

Lastly, we note issues to be addressed in future research. The current study adopted a within-subject design, and all the child participants completed the Baseline Condition in the first testing session and the Focused *every* Condition in the second session. We intended to test the Baseline Condition without prior exposure to the critical experimental discourse in order to compare results from Turkish-speaking children to those reported for other language groups in previous studies. Due to practical challenges, it was impossible to recruit a larger sample of child participants to counterbalance the session order. Given this, our findings could instead reflect effects of general learning. This raises a question regarding whether the improved accuracy for the second session was truly due to the crucial experimental manipulation, or preceding exposure to the Baseline Condition in the first session, reflecting some sort of carryover effect.

To our knowledge, Minai et al. (2012) is the only study in the universal quantification literature that adopted a within-subject design in a blocked single session. Minai et al. (2012) revealed that exposure to the extra-object condition in the first block did not affect comprehension patterns in the second block. Aravind et al. (2017) conducted a longitudinal study, where children were also tested repeatedly with the same extra-object condition over ages 4 to 7. Interestingly, their longitudinal data revealed *increased* rates of q-spreading across datapoints, a finding opposing to facilitative adaptation that calls for explanation beyond the current scope. The only study which revealed data suggestive of a carryover effect is Minai et al. (2025), but their design was different from ours in that participants were presented with only one syntactic condition, corresponding to our Baseline Condition, and three prosodic conditions, and a group of child participants shifted from q-spreading to adult-like comprehension *during the single testing session*. Hence, while not being able to tease apart the two possibilities mentioned above based on the current findings alone, previous q-spreading studies reveal little evidence of a carryover effect *over time*, where previous exposure to the extra-object experimental setting in the first session facilitates children’s successful avoidance of q-spreading in the subsequent session scheduled on a different day. Future research using an alternative blocking approach may help clarify the extent to which the utilization of morphologically-marked focus in the comprehension of quantified sentences is impacted by factors such as block order.

Recall that the current study also revealed variable response patterns across conditions. Interestingly, some children showed more adult-like responses in the second session. Findings revealing various response patterns are consistent with other findings suggesting that there is a range of individual differences among children with respect to the degree to which they are prone to exhibit q-spreading (e.g., Minai et al., 2012, 2025). It has been an open question regarding why some children consistently exhibit q-spreading, regardless of the conditions^4^. In the current study, we did not include any direct measure to assess children’s individual differences. While the lack of this information does not allow us to generate a compelling explanation, we speculate that our focus manipulation was ineffective for the children who adhere to q-spreading robustly. Future research is therefore necessary to investigate what factors may contribute to the variability.

We also acknowledge that it is useful to include another baseline condition, object focus alongside a universally-quantified subject. This new baseline condition would allow us to further examine the effect of attentional focus marking, by testing whether the focalized object may serve as an additional cue toward q-spreading, on top of the presence of the extra object in the visual materials which has been argued to facilitate q-spreading (e.g., Minai et al., 2012, 2025; Sugisaki and Isobe, 2001). If both cues cumulatively bias children to commit to q-spreading, then higher rates of q-spreading would be predicted for this object focus condition than for a baseline condition without focus marking. In the current study, we did not include such a condition as we primarily investigated whether subject focus marking could disengage children from paying attention to the extra object. We acknowledge that including this condition is an issue to be addressed in future research.^5^

## 8 Conclusion

The current study examined young children’s awareness of focus marking in comprehension, examining sensitivity to morphologically-marked focus in Turkish. When the QP *mI,* which typically appears at the end of a question sentence, is attached to a non-predicate constituent in the sentence, narrow attentional focus obtains on that constituent. We used Turkish question sentences involving a universal quantifier, which are known to elicit an erroneous interpretation from preschool children when presented with an “extra object” picture, known as q-spreading. We manipulated the placement of the QP in the Focused *every* Condition, in which the QP focuses the universally-quantified subject, yielding a narrow-focus reading on par with a cleft sentence (e.g., “Is it every child that is holding a balloon?”), and the Baseline Condition, in which the QP appears at the end of the question sentence and is interpreted as a simple Yes/No question with a broad-focus reading (e.g., “Is every child holding a balloon?”). Our results reveal that rates of q-spreading were significantly lower for the Focused *every* Condition, where the attentional focus was marked on the universally-quantified subject, than for the Baseline Condition. Findings suggested that children incorporated morphological focus marking and manipulated attentional focus, drawing attention to the subject and away from the object associated with q-spreading errors. These are important findings demonstrating children’s early awareness of morphological focus marking. The current study also expanded the range of linguistic devices marking focus, rather than devices like focus operators, prosody and clefting examined by previous studies, and provided evidence consistent with the “full competence” view in the domain of focus.

## Data Availability

The original contributions presented in the study are included in the article/supplementary material, further inquiries can be directed to the corresponding author.

## Footnotes

1 We first fit a model including a by-subject random slope for Condition (1 + Condition | Subject). However, this model gave a singular fit warning, indicating that the random-effects structure was overparameterized relative to the information available in the data, potentially due to two within-subject conditions and a limited number of trials per cell, the by-subject slope variance was estimated at the boundary of the parameter space. We simplified the random-effects structure to a by-subject random intercept, which converged without giving a singular fit warning.

2 We thank an anonymous reviewer for bringing this point to our attention.

3 We are grateful to an anonymous reviewer for pointing this out.

4 We thank an anonymous reviewer for raising this point.

5 We appreciate an anonymous reviewer raising this point.
