# Supplementary material for: Examining children’s sensitivity to morphologically-marked focus in the comprehension of question sentences: evidence from quantifier comprehension in child Turkish
Source: Front Hum Neurosci. 2026 Oct 14;20:1914200. doi: 10.3389/fnhum.2026.1914200 (PMC13714967; doi:10.3389/fnhum.2026.1914200)
Supplement: Supplementary file 1 [file Data_Sheet_1.pdf]

Data analysis scripts

2026-09-03

```
setwd("")
newdata = read.csv("newdata.csv", header = TRUE, stringsAsFactors = TRUE)
str(newdata)

## 'data.frame':    864 obs. of  14 variables:
##  $ Subject    : Factor w/ 36 levels "CH001","CH002",..: 1 1 1 1 1 1 1 1 1
1 ...
##  $ Age        : num  4.4 4.4 4.4 4.4 4.4 4.4 4.4 4.4 4.4 4.4 ...
##  $ Gender     : Factor w/ 2 levels "Female","Male": 1 1 1 1 1 1 1 1 1 1
...
##  $ Group      : Factor w/ 1 level "Child": 1 1 1 1 1 1 1 1 1 1 ...
##  $ Session    : int  1 1 1 1 1 1 1 1 1 1 ...
##  $ Item       : Factor w/ 12 levels "1","10","2","3",..: 11 11 1 3 4 5 6 7
8 9 ...
##  $ TrialOrder : int  1 2 3 4 5 6 7 8 9 10 ...
##  $ Type       : Factor w/ 3 levels "Experimental",..: 3 3 1 2 1 2 1 2 1 2
...
##  $ Condition  : Factor w/ 3 levels "\"Extra-object\"",..: 1 1 1 1 1 1 1 1
1 1 ...
##  $ List       : int  1 1 1 1 1 1 1 1 1 1 ...
##  $ Experiment : int  1 1 1 1 1 1 1 1 1 1 ...
##  $ Response   : Factor w/ 2 levels "Evet","Hayır": 1 2 1 2 2 1 1 2 2 1 ...
##  $ Explanation: Factor w/ 531 levels "3 çocuk da kitap okuyor ama bir
kitap boşta",..: 26 12 223 214 111 224 220 153 149 375 ...
##  $ Accuracy   : int  1 1 1 1 0 1 1 1 0 1 ...

levels(newdata$Condition)[levels(newdata$Condition) == "\"Extra-object\""] <-
"Baseline"
newdata$Experiment <- factor(newdata$Experiment)



Experimental_only <- newdata[newdata$Type== "Experimental",]

options(scipen=999)

minorcolors1 <- c("darkkhaki", "indianred")
minorcolors2 <- c("darkkhaki", "cadetblue")

Experiment1_only <- Experimental_only[Experimental_only$Experiment== "1",]
str(Experiment1_only)

## 'data.frame':    180 obs. of  14 variables:
##  $ Subject    : Factor w/ 36 levels "CH001","CH002",..: 1 1 1 1 1 1 1 1 1
1 ...
##  $ Age        : num  4.4 4.4 4.4 4.4 4.4 4.4 4.4 4.4 4.4 4.4 ...
##  $ Gender     : Factor w/ 2 levels "Female","Male": 1 1 1 1 1 1 1 1 1 1
```

```
...
## $ Group      : Factor w/ 1 level "Child": 1 1 1 1 1 1 1 1 1 1 ...
## $ Session    : int  1 1 1 1 1 2 2 2 2 2 ...
## $ Item       : Factor w/ 12 levels "1","10","2","3",..: 1 4 6 8 10 10 8 6
4 1 ...
## $ TrialOrder : int  3 5 7 9 11 4 6 8 10 12 ...
## $ Type       : Factor w/ 3 levels "Experimental",..: 1 1 1 1 1 1 1 1 1 1
...
## $ Condition  : Factor w/ 3 levels "Baseline","Focused every",..: 1 1 1 1
1 2 2 2 2 2 ...
## $ List       : int  1 1 1 1 1 2 2 2 2 2 ...
## $ Experiment : Factor w/ 2 levels "1","2": 1 1 1 1 1 1 1 1 1 1 ...
## $ Response   : Factor w/ 2 levels "Evet","Hayır": 1 2 1 2 2 1 1 2 2 2 ...
## $ Explanation: Factor w/ 531 levels "3 çocuk da kitap okuyor ama bir
kitap boşta",..: 223 111 220 149 181 327 513 181 159 175 ...
## $ Accuracy   : int  1 0 1 0 0 1 1 0 0 0 ...
```

Step-by-step analysis

```
##############################################################################
###
# STEP 0 — Packages and objects
##############################################################################
###

library(dplyr)

##
## Attaching package: 'dplyr'

## The following objects are masked from 'package:stats':
##
##     filter, lag

## The following objects are masked from 'package:base':
##
##     intersect, setdiff, setequal, union

library(ggplot2)
library(lme4)

## Loading required package: Matrix

library(emmeans)

## Welcome to emmeans.
## Caution: You lose important information if you filter this package's
results.
## See '? untidy'

library(brms)
```

```
## Loading required package: Rcpp

## Loading 'brms' package (version 2.21.0). Useful instructions
## can be found by typing help('brms'). A more detailed introduction
## to the package is available through vignette('brms_overview').

##
## Attaching package: 'brms'

## The following object is masked from 'package:lme4':
##
##     ngrps

## The following object is masked from 'package:stats':
##
##     ar

library(bayestestR)

## Warning: package 'bayestestR' was built under R version 4.4.1

library(posterior)

## This is posterior version 1.6.0

##
## Attaching package: 'posterior'

## The following objects are masked from 'package:stats':
##
##     mad, sd, var

## The following objects are masked from 'package:base':
##
##     %in%, match

# Create an output folder so fitted models can be cached
dir.create("fits", showWarnings = FALSE)

# Set once; used throughout
options(mc.cores = 4)

############################################################################
###
# STEP 1 — Data integrity
############################################################################
###

str(Experiment1_only)

## 'data.frame':    180 obs. of  14 variables:
##  $ Subject    : Factor w/ 36 levels "CH001","CH002",..: 1 1 1 1 1 1 1 1 1
```

```
1 ...
##  $ Age        : num  4.4 4.4 4.4 4.4 4.4 4.4 4.4 4.4 4.4 4.4 ...
##  $ Gender     : Factor w/ 2 levels "Female","Male": 1 1 1 1 1 1 1 1 1 1
...
##  $ Group      : Factor w/ 1 level "Child": 1 1 1 1 1 1 1 1 1 1 ...
##  $ Session    : int  1 1 1 1 1 2 2 2 2 2 ...
##  $ Item       : Factor w/ 12 levels "1","10","2","3",..: 1 4 6 8 10 10 8 6
4 1 ...
##  $ TrialOrder : int  3 5 7 9 11 4 6 8 10 12 ...
##  $ Type       : Factor w/ 3 levels "Experimental",..: 1 1 1 1 1 1 1 1 1 1
...
##  $ Condition  : Factor w/ 3 levels "Baseline","Focused every",..: 1 1 1 1
1 2 2 2 2 2 ...
##  $ List       : int  1 1 1 1 1 2 2 2 2 2 ...
##  $ Experiment : Factor w/ 2 levels "1","2": 1 1 1 1 1 1 1 1 1 1 ...
##  $ Response   : Factor w/ 2 levels "Evet","Hayır": 1 2 1 2 2 1 1 2 2 2 ...
##  $ Explanation: Factor w/ 531 levels "3 çocuk da kitap okuyor ama bir
kitap boşta",..: 223 111 220 149 181 327 513 181 159 175 ...
##  $ Accuracy   : int  1 0 1 0 0 1 1 0 0 0 ...

# DV must be 0/1 only
table(Experiment1_only$Accuracy, useNA = "ifany")

## 
##   0   1 
##  67 113

# Total observations, subjects, items
nrow(Experiment1_only)

## [1] 180

n_distinct(Experiment1_only$Subject)

## [1] 18

n_distinct(Experiment1_only$Item)

## [1] 5

# Missing data
colSums(is.na(Experiment1_only[, c("Accuracy", "Condition", "Subject",
"Item")]))

##  Accuracy Condition   Subject      Item 
##         0         0         0         0

#############################################################################
###
# STEP 2 — Design structure
#############################################################################
###
```

```r
# Trials per subject per condition --> should be 5 in every cell if fully within
table(Experiment1_only$Subject, Experiment1_only$Condition)

##
##         Baseline Focused every QUD
##   CH001        5             5   0
##   CH002        0             0   0
##   CH003        0             0   0
##   CH004        5             5   0
##   CH005        5             5   0
##   CH006        0             0   0
##   CH007        5             5   0
##   CH008        0             0   0
##   CH009        5             5   0
##   CH010        0             0   0
##   CH011        5             5   0
##   CH012        0             0   0
##   CH013        5             5   0
##   CH014        5             5   0
##   CH015        5             5   0
##   CH016        5             5   0
##   CH017        0             0   0
##   CH018        0             0   0
##   CH019        5             5   0
##   CH020        0             0   0
##   CH021        0             0   0
##   CH022        5             5   0
##   CH023        0             0   0
##   CH024        0             0   0
##   CH025        5             5   0
##   CH026        0             0   0
##   CH027        0             0   0
##   CH028        5             5   0
##   CH029        0             0   0
##   CH030        5             5   0
##   CH031        5             5   0
##   CH032        0             0   0
##   CH033        0             0   0
##   CH034        0             0   0
##   CH035        5             5   0
##   CH036        5             5   0

# Is each Item seen in both conditions, or does each Item belong to one?
table(Experiment1_only$Item, Experiment1_only$Condition)

##
##             Baseline Focused every QUD
##   1               18            18   0
```

```
##   10                0              0   0
##   2                 0              0   0
##   3                18             18   0
##   4                 0              0   0
##   5                18             18   0
##   6                 0              0   0
##   7                18             18   0
##   8                 0              0   0
##   9                18             18   0
##   Practice          0              0   0
##   Practice          0              0   0
```

```
#############################################################################
###
# STEP 3 — Fix factor coding explicitly
#############################################################################
###

table(Experiment1_only$Condition, useNA = "ifany")
```

```
##
##      Baseline Focused every           QUD
##            90            90             0
```

```
Experiment1_only$Condition <- droplevels(Experiment1_only$Condition)
Experiment1_only$Condition <- relevel(Experiment1_only$Condition, ref =
"Baseline")
levels(Experiment1_only$Condition)   # "Baseline" "Focused every"
```

```
## [1] "Baseline"      "Focused every"
```

```
Experiment1_only$Subject <- droplevels(as.factor(Experiment1_only$Subject))
Experiment1_only$Item    <- droplevels(as.factor(Experiment1_only$Item))

nlevels(Experiment1_only$Subject)
```

```
## [1] 18
```

```
nlevels(Experiment1_only$Item)
```

```
## [1] 5
```

```
#############################################################################
###
# STEP 4 — Descriptives
#############################################################################
###

# 4a. Trial-level
desc_trial <- Experiment1_only %>%
  group_by(Condition) %>%
  summarise(
```

```
    n_trials = n(),
    k        = sum(Accuracy),
    prop     = k / n_trials,
    se_naive = sqrt(prop * (1 - prop) / n_trials),
    .groups  = "drop"
  )
desc_trial

## # A tibble: 2 × 5
##   Condition     n_trials     k  prop se_naive
##   <fct>            <int> <int> <dbl>    <dbl>
## 1 Baseline            90    52 0.578   0.0521
## 2 Focused every       90    61 0.678   0.0493

# 4b. By-subject proportions, then between-subject SE
subj_prop <- Experiment1_only %>%
  group_by(Subject, Condition) %>%
  summarise(prop = mean(Accuracy), n_trials = n(), .groups = "drop")

desc_subj <- subj_prop %>%
  group_by(Condition) %>%
  summarise(
    n_subjects = n(),
    mean_prop  = mean(prop),
    sd_prop    = sd(prop),
    se_prop    = sd(prop) / sqrt(n()),
    .groups    = "drop"
  )
desc_subj

## # A tibble: 2 × 5
##   Condition     n_subjects mean_prop sd_prop se_prop
##   <fct>              <int>     <dbl>   <dbl>   <dbl>
## 1 Baseline              18     0.578   0.460   0.108
## 2 Focused every         18     0.678   0.429   0.101

#############################################################################
###
# STEP 5 — Separation diagnostic
#############################################################################
###

subj_wide <- subj_prop %>%
  dplyr::mutate(pattern = dplyr::case_when(
    prop == 1 ~ "ceiling",
    prop == 0 ~ "floor",
    TRUE      ~ "mixed"
  )) %>%
  dplyr::select(Subject, Condition, prop, pattern) %>%
  tidyr::pivot_wider(
    names_from  = Condition,
```

```
    values_from = c(prop, pattern)
  )
print(as.data.frame(subj_wide))
```

```
##    Subject prop_Baseline prop_Focused every pattern_Baseline
## 1    CH001           0.4                0.4            mixed
## 2    CH004           0.0                0.2            floor
## 3    CH005           0.0                0.4            floor
## 4    CH007           1.0                1.0          ceiling
## 5    CH009           0.0                0.0            floor
## 6    CH011           1.0                1.0          ceiling
## 7    CH013           0.0                0.0            floor
## 8    CH014           1.0                1.0          ceiling
## 9    CH015           1.0                1.0          ceiling
## 10   CH016           1.0                1.0          ceiling
## 11   CH019           0.2                0.2            mixed
## 12   CH022           1.0                1.0          ceiling
## 13   CH025           1.0                1.0          ceiling
## 14   CH028           0.2                1.0            mixed
## 15   CH030           0.0                0.0            floor
## 16   CH031           1.0                1.0          ceiling
## 17   CH035           0.6                1.0            mixed
## 18   CH036           1.0                1.0          ceiling
##    pattern_Focused every
## 1                  mixed
## 2                  mixed
## 3                  mixed
## 4                ceiling
## 5                  floor
## 6                ceiling
## 7                  floor
## 8                ceiling
## 9                ceiling
## 10               ceiling
## 11                 mixed
## 12               ceiling
## 13               ceiling
## 14               ceiling
## 15                 floor
## 16               ceiling
## 17               ceiling
## 18               ceiling
```

```
# Cross-tabulation of response pattern by condition
sep_table <- subj_prop %>%
  dplyr::mutate(pattern = dplyr::case_when(
    prop == 1 ~ "ceiling",
    prop == 0 ~ "floor",
    TRUE      ~ "mixed"
  )) %>%
```

```
  dplyr::select(Subject, Condition, pattern) %>%
  tidyr::pivot_wider(names_from = Condition, values_from = pattern) %>%
  dplyr::count(dplyr::pick(-Subject))
print(as.data.frame(sep_table))

##   Baseline Focused every n
## 1  ceiling       ceiling 9
## 2    floor         floor 3
## 3    floor         mixed 2
## 4    mixed       ceiling 2
## 5    mixed         mixed 2

# How many subjects are extreme in BOTH conditions?
n_extreme_both <- subj_prop %>%
  dplyr::mutate(extreme = prop %in% c(0, 1)) %>%
  dplyr::group_by(Subject) %>%
  dplyr::summarise(both = all(extreme), .groups = "drop") %>%
  dplyr::summarise(n = sum(both)) %>%
  dplyr::pull(n)
n_extreme_both

## [1] 12

#############################################################################
###
# STEP 6 — glmer models
#############################################################################
###

m_null <- glmer(Accuracy ~ 1 + (1 | Subject) + (1 | Item),
                data = Experiment1_only,
                family = binomial(link = "logit"),
                control = glmerControl(optimizer = "bobyqa"))

m_full <- glmer(Accuracy ~ Condition + (1 | Subject) + (1 | Item),
                data = Experiment1_only,
                family = binomial(link = "logit"),
                control = glmerControl(optimizer = "bobyqa"))

summary(m_full)

## Generalized linear mixed model fit by maximum likelihood (Laplace
##   Approximation) [glmerMod]
##  Family: binomial  ( logit )
## Formula: Accuracy ~ Condition + (1 | Subject) + (1 | Item)
##    Data: Experiment1_only
## Control: glmerControl(optimizer = "bobyqa")
##
##      AIC      BIC   logLik deviance df.resid
##    111.9    124.7    -52.0    103.9      176
##
```

```
## Scaled residuals:
##      Min       1Q   Median       3Q      Max 
## -1.58949 -0.10070  0.00539  0.01552  2.87555 
## 
## Random effects:
##  Groups  Name        Variance Std.Dev.
##  Subject (Intercept) 175.667  13.2539 
##  Item    (Intercept)   0.867   0.9311 
## Number of obs: 180, groups:  Subject, 18; Item, 5
## 
## Fixed effects:
##                          Estimate Std. Error z value Pr(>|z|)    
## (Intercept)                8.3956     2.4410   3.439 0.000583 ***
## ConditionFocused every     2.1167     0.8121   2.607 0.009143 ** 
## ---
## Signif. codes:  0 '***' 0.001 '**' 0.01 '*' 0.05 '.' 0.1 ' ' 1
## 
## Correlation of Fixed Effects:
##             (Intr)
## CndtnFcsdev 0.151
```

```
# Likelihood ratio test
lrt <- anova(m_null, m_full)
lrt
```

```
## Data: Experiment1_only
## Models:
## m_null: Accuracy ~ 1 + (1 | Subject) + (1 | Item)
## m_full: Accuracy ~ Condition + (1 | Subject) + (1 | Item)
##        npar    AIC    BIC  logLik deviance  Chisq Df Pr(>Chisq)   
## m_null    3 118.64 128.22 -56.319   112.64                        
## m_full    4 111.91 124.68 -51.956   103.91 8.7277  1   0.003134 **
## ---
## Signif. codes:  0 '***' 0.001 '**' 0.01 '*' 0.05 '.' 0.1 ' ' 1
```

```
#############################################################################
###
# STEP 7 — Instability diagnostics for the ML fit
# >>> This is the evidence that justifies switching to the Bayesian model
#############################################################################
###

# Random effect SDs -- a huge subject SD is the separation signature
print(VarCorr(m_full), comp = "Std.Dev.")
```

```
##  Groups  Name        Std.Dev.
##  Subject (Intercept) 13.25394
##  Item    (Intercept)  0.93114
```

```
# Singular fit?
isSingular(m_full)
```

```
## [1] FALSE

isSingular(m_null)

## [1] FALSE

# Any convergence warnings stored on the fit?
m_full@optinfo$conv$lme4$messages

## NULL

#############################################################################
###
# STEP 8 — Model-based means from glmer (for comparison with brms later)
#############################################################################
###

emm_glmer <- emmeans(m_full, ~ Condition, type = "response")
emm_glmer

##  Condition        prob          SE  df asymp.LCL asymp.UCL
##  Baseline      0.99977 0.000551058 Inf   0.97369         1
##  Focused every 0.99997 0.000073065 Inf   0.99476         1
##
## Confidence level used: 0.95
## Intervals are back-transformed from the logit scale

pairs(emm_glmer)   # this is an ODDS RATIO, not a probability difference

##  contrast                 odds.ratio     SE  df null z.ratio p.value
##  Baseline / Focused every       0.12 0.0978 Inf    1  -2.607  0.0091
##
## Tests are performed on the log odds ratio scale

#############################################################################
###
# STEP 9 — brms priors (matched across models, no centering mismatch)
#############################################################################
###

# Inspect which priors the model expects before setting them
get_prior(Accuracy ~ 0 + Intercept + Condition + (1 | Subject) + (1 | Item),
          data = Experiment1_only, family = bernoulli())

##                  prior class                  coef   group resp dpar nlpar
lb ub
##                 (flat)     b
##                 (flat)     b ConditionFocusedevery
##                 (flat)     b             Intercept
##  student_t(3, 0, 2.5)    sd
0
##  student_t(3, 0, 2.5)    sd                           Item
```

```
0
##  student_t(3, 0, 2.5)    sd              Intercept    Item
0
##  student_t(3, 0, 2.5)    sd                        Subject
0
##  student_t(3, 0, 2.5)    sd              Intercept Subject
0
##        source
##       default
##  (vectorized)
##  (vectorized)
##       default
##  (vectorized)
##  (vectorized)
##  (vectorized)
##  (vectorized)

priors_full <- c(
  prior(normal(0, 2),        class = "b"),                     # Condition
effect
  prior(normal(0, 2),        class = "b", coef = "Intercept"), # explicit
intercept
  prior(student_t(3, 0, 2),  class = "sd")
)

priors_null <- c(
  prior(normal(0, 2),        class = "b", coef = "Intercept"),
  prior(student_t(3, 0, 2),  class = "sd")
)

unlink("fits", recursive = TRUE); dir.create("fits")


#############################################################################
###
# STEP 10 — Fit the two Bayesian models
#############################################################################
###

fit_full <- brm(
  Accuracy ~ 0 + Intercept + Condition + (1 | Subject) + (1 | Item),
  data      = Experiment1_only,
  family    = bernoulli(link = "logit"),
  prior     = priors_full,
  chains    = 4,
  iter      = 25000,
  warmup    = 5000,
  control   = list(adapt_delta = 0.99, max_treedepth = 12),
  save_pars = save_pars(all = TRUE),
  seed      = 123,
```

```
  file      = "fits/fit_full"
)

## Compiling Stan program...

## Trying to compile a simple C file

## Running /Library/Frameworks/R.framework/Resources/bin/R CMD SHLIB foo.c
## using C compiler: 'Apple clang version 16.0.0 (clang-1600.0.26.4)'
## using SDK: 'MacOSX15.1.sdk'
## clang -arch arm64 -I"/Library/Frameworks/R.framework/Resources/include" -
DNDEBUG   -I"/Library/Frameworks/R.framework/Versions/4.4-
arm64/Resources/library/Rcpp/include/"  -
I"/Library/Frameworks/R.framework/Versions/4.4-
arm64/Resources/library/RcppEigen/include/"  -
I"/Library/Frameworks/R.framework/Versions/4.4-
arm64/Resources/library/RcppEigen/include/unsupported"  -
I"/Library/Frameworks/R.framework/Versions/4.4-
arm64/Resources/library/BH/include" -
I"/Library/Frameworks/R.framework/Versions/4.4-
arm64/Resources/library/StanHeaders/include/src/"  -
I"/Library/Frameworks/R.framework/Versions/4.4-
arm64/Resources/library/StanHeaders/include/"  -
I"/Library/Frameworks/R.framework/Versions/4.4-
arm64/Resources/library/RcppParallel/include/"  -
I"/Library/Frameworks/R.framework/Versions/4.4-
arm64/Resources/library/rstan/include" -DEIGEN_NO_DEBUG  -
DBOOST_DISABLE_ASSERTS  -DBOOST_PENDING_INTEGER_LOG2_HPP  -DSTAN_THREADS  -
DUSE_STANC3 -DSTRICT_R_HEADERS  -DBOOST_PHOENIX_NO_VARIADIC_EXPRESSION  -
D_HAS_AUTO_PTR_ETC=0  -include '/Library/Frameworks/R.framework/Versions/4.4-
arm64/Resources/library/StanHeaders/include/stan/math/prim/fun/Eigen.hpp'  -
D_REENTRANT -DRCPP_PARALLEL_USE_TBB=1   -I/opt/R/arm64/include    -fPIC  -
falign-functions=64 -Wall -g -O2  -c foo.c -o foo.o
## In file included from <built-in>:1:
## In file included from /Library/Frameworks/R.framework/Versions/4.4-
arm64/Resources/library/StanHeaders/include/stan/math/prim/fun/Eigen.hpp:22:
## In file included from /Library/Frameworks/R.framework/Versions/4.4-
arm64/Resources/library/RcppEigen/include/Eigen/Dense:1:
## In file included from /Library/Frameworks/R.framework/Versions/4.4-
arm64/Resources/library/RcppEigen/include/Eigen/Core:19:
## /Library/Frameworks/R.framework/Versions/4.4-
arm64/Resources/library/RcppEigen/include/Eigen/src/Core/util/Macros.h:679:10
: fatal error: 'cmath' file not found
##   679 | #include <cmath>
##       |          ^~~~~~~
## 1 error generated.
## make: *** [foo.o] Error 1

## Start sampling
```

```r
fit_null <- brm(
  Accuracy ~ 0 + Intercept + (1 | Subject) + (1 | Item),
  data      = Experiment1_only,
  family    = bernoulli(link = "logit"),
  prior     = priors_null,
  chains    = 4,
  iter      = 25000,
  warmup    = 5000,
  control   = list(adapt_delta = 0.99, max_treedepth = 12),
  save_pars = save_pars(all = TRUE),
  seed      = 123,
  file      = "fits/fit_null"
)
```

```
## Compiling Stan program...
## Trying to compile a simple C file

## Running /Library/Frameworks/R.framework/Resources/bin/R CMD SHLIB foo.c
## using C compiler: ‘Apple clang version 16.0.0 (clang-1600.0.26.4)’
## using SDK: ‘MacOSX15.1.sdk’
## clang -arch arm64 -I"/Library/Frameworks/R.framework/Resources/include" -
DNDEBUG   -I"/Library/Frameworks/R.framework/Versions/4.4-
arm64/Resources/library/Rcpp/include/"  -
I"/Library/Frameworks/R.framework/Versions/4.4-
arm64/Resources/library/RcppEigen/include/"  -
I"/Library/Frameworks/R.framework/Versions/4.4-
arm64/Resources/library/RcppEigen/include/unsupported"  -
I"/Library/Frameworks/R.framework/Versions/4.4-
arm64/Resources/library/BH/include" -
I"/Library/Frameworks/R.framework/Versions/4.4-
arm64/Resources/library/StanHeaders/include/src/"  -
I"/Library/Frameworks/R.framework/Versions/4.4-
arm64/Resources/library/StanHeaders/include/"  -
I"/Library/Frameworks/R.framework/Versions/4.4-
arm64/Resources/library/RcppParallel/include/"  -
I"/Library/Frameworks/R.framework/Versions/4.4-
arm64/Resources/library/rstan/include" -DEIGEN_NO_DEBUG  -
DBOOST_DISABLE_ASSERTS  -DBOOST_PENDING_INTEGER_LOG2_HPP  -DSTAN_THREADS  -
DUSE_STANC3 -DSTRICT_R_HEADERS  -DBOOST_PHOENIX_NO_VARIADIC_EXPRESSION  -
D_HAS_AUTO_PTR_ETC=0  -include '/Library/Frameworks/R.framework/Versions/4.4-
arm64/Resources/library/StanHeaders/include/stan/math/prim/fun/Eigen.hpp'  -
D_REENTRANT -DRCPP_PARALLEL_USE_TBB=1   -I/opt/R/arm64/include    -fPIC  -
falign-functions=64 -Wall -g -O2  -c foo.c -o foo.o
## In file included from <built-in>:1:
## In file included from /Library/Frameworks/R.framework/Versions/4.4-
arm64/Resources/library/StanHeaders/include/stan/math/prim/fun/Eigen.hpp:22:
## In file included from /Library/Frameworks/R.framework/Versions/4.4-
arm64/Resources/library/RcppEigen/include/Eigen/Dense:1:
## In file included from /Library/Frameworks/R.framework/Versions/4.4-
arm64/Resources/library/RcppEigen/include/Eigen/Core:19:
```

```
## /Library/Frameworks/R.framework/Versions/4.4-arm64/Resources/library/RcppEigen/include/Eigen/src/Core/util/Macros.h:679:10
: fatal error: 'cmath' file not found
##   679 | #include <cmath>
##       |          ^~~~~~~
## 1 error generated.
## make: *** [foo.o] Error 1

## Start sampling

summary(fit_full)

##  Family: bernoulli
##   Links: mu = logit
## Formula: Accuracy ~ 0 + Intercept + Condition + (1 | Subject) + (1 | Item)
##    Data: Experiment1_only (Number of observations: 180)
##   Draws: 4 chains, each with iter = 25000; warmup = 5000; thin = 1;
##          total post-warmup draws = 80000
##
## Multilevel Hyperparameters:
## ~Item (Number of levels: 5)
##               Estimate Est.Error l-95% CI u-95% CI Rhat Bulk_ESS Tail_ESS
## sd(Intercept)     1.00      0.69     0.07     2.68 1.00    25594    26903
##
## ~Subject (Number of levels: 18)
##               Estimate Est.Error l-95% CI u-95% CI Rhat Bulk_ESS Tail_ESS
## sd(Intercept)     6.55      2.24     3.51    12.13 1.00    25208    38699
##
## Regression Coefficients:
##                       Estimate Est.Error l-95% CI u-95% CI Rhat Bulk_ESS
## Intercept                 0.98      1.33    -1.65     3.64 1.00    20204
## ConditionFocusedevery     1.74      0.68     0.48     3.17 1.00    76848
##                       Tail_ESS
## Intercept                32769
## ConditionFocusedevery    52702
##
## Draws were sampled using sampling(NUTS). For each parameter, Bulk_ESS
## and Tail_ESS are effective sample size measures, and Rhat is the potential
## scale reduction factor on split chains (at convergence, Rhat = 1).

#############################################################################
###
# STEP 11 — Convergence diagnostics
#############################################################################
###

# R-hat and effective sample sizes
summarise_draws(as_draws_df(fit_full), "rhat", "ess_bulk", "ess_tail") |>
  as.data.frame() |> head(10)
```

```
##                        variable     rhat ess_bulk ess_tail
## 1                   b_Intercept 1.000178 20203.82 32768.73
## 2     b_ConditionFocusedevery 1.000167 76847.64 52701.87
## 3          sd_Item__Intercept 1.000051 25594.49 26902.70
## 4       sd_Subject__Intercept 1.000054 25208.04 38699.21
## 5         r_Item[1,Intercept] 1.000024 59938.70 62357.60
## 6         r_Item[3,Intercept] 1.000021 58129.66 63756.55
## 7         r_Item[5,Intercept] 1.000083 56411.67 65024.78
## 8         r_Item[7,Intercept] 1.000008 64176.34 62399.42
## 9         r_Item[9,Intercept] 1.000011 36425.50 57515.01
## 10 r_Subject[CH001,Intercept] 1.000040 22390.84 37622.72

# Divergent transitions and treedepth saturation
np <- nuts_params(fit_full)
table(np$Parameter[np$Value > 0 & np$Parameter == "divergent__"])

##
## accept_stat__    stepsize__   treedepth__  n_leapfrog__   divergent__
##             0             0             0             0             0
##      energy__
##             0

sum(subset(np, Parameter == "divergent__")$Value)

## [1] 0

# Visual checks
plot(fit_full)
```

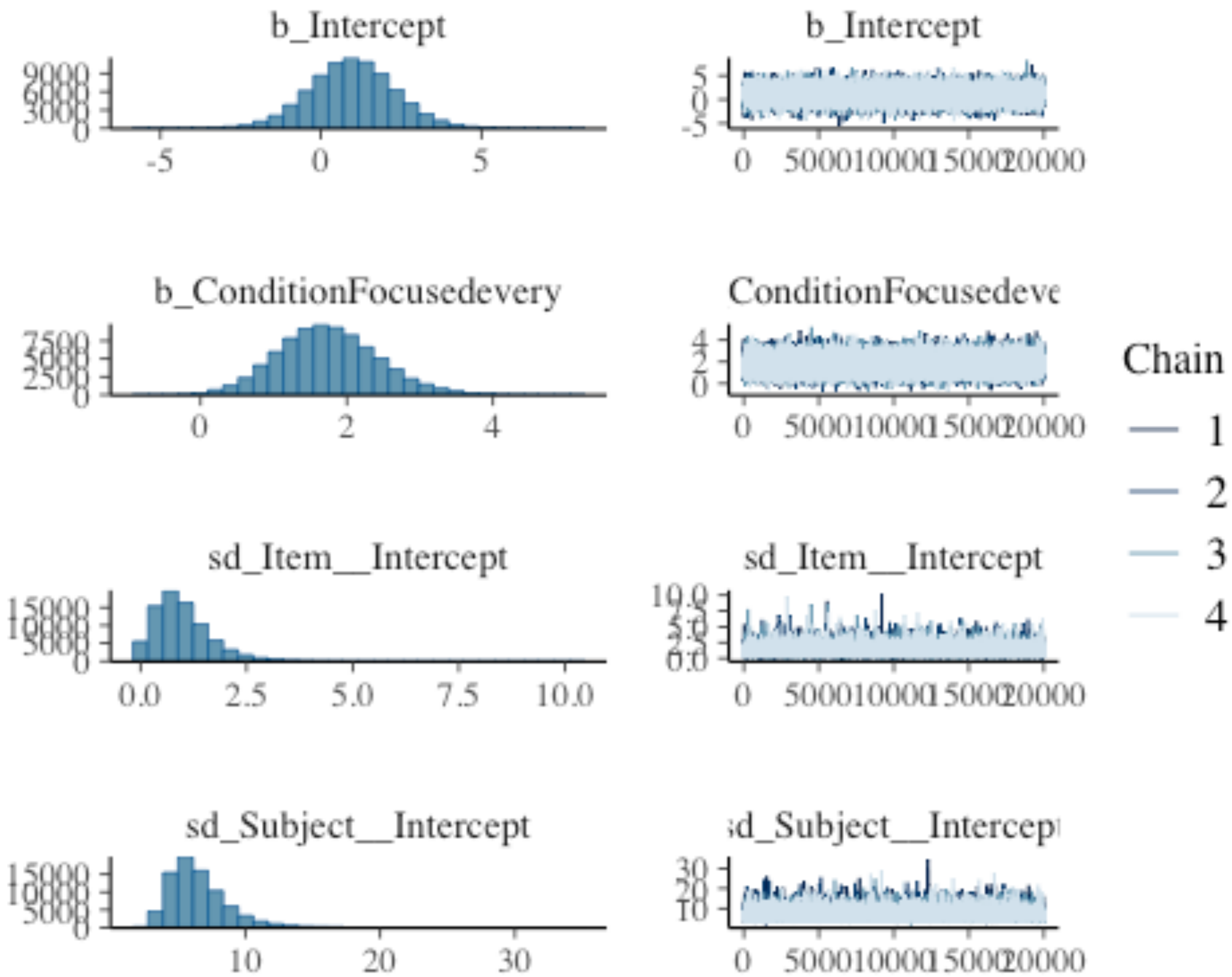


```
pp_check(fit_full, ndraws = 100)
```

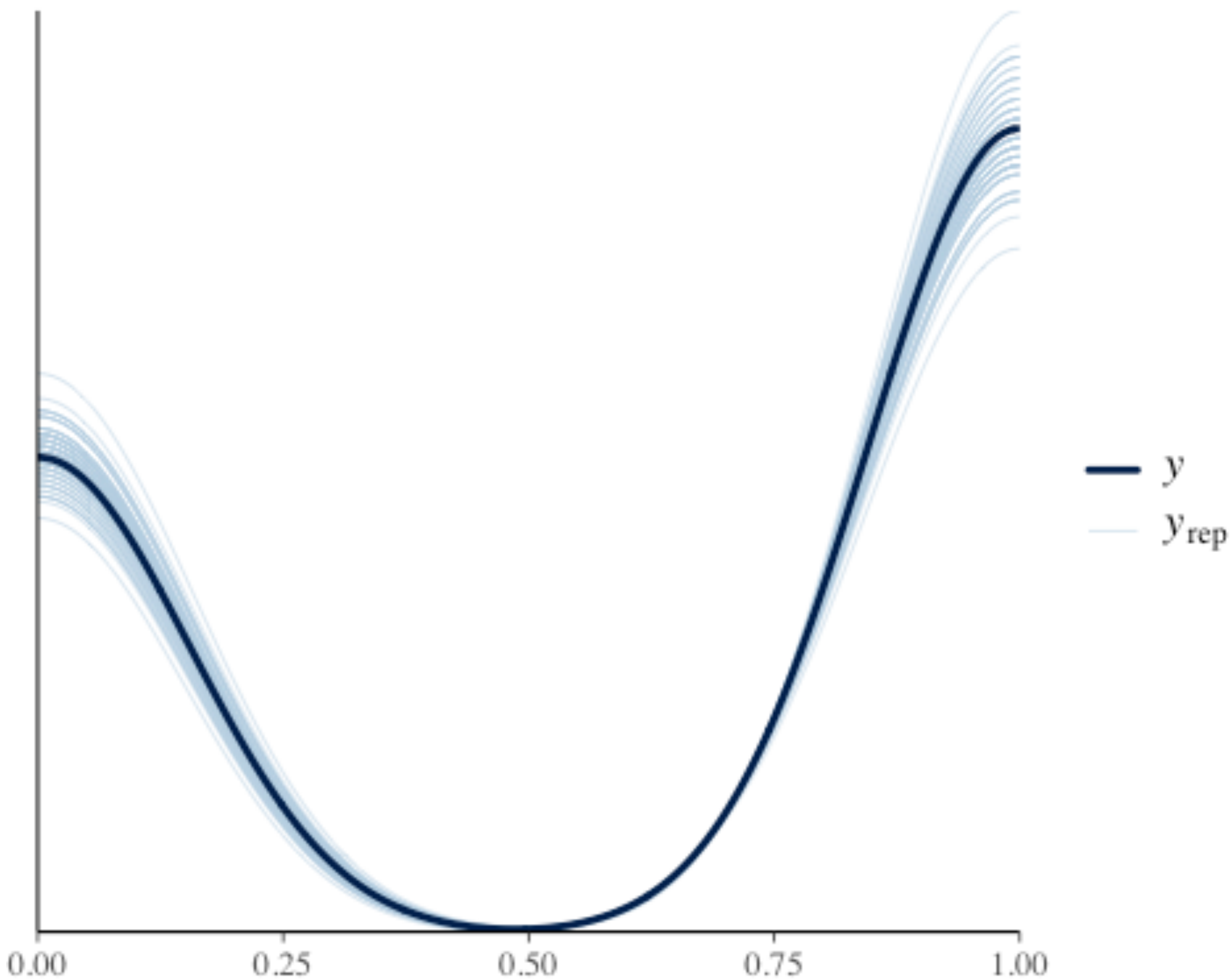


```
################################################################################
###
# STEP 12 — Posterior summaries
################################################################################
###

# 13a. The Condition coefficient (log-odds) -- EDIT name to match your level
fixef(fit_full)

##                         Estimate Est.Error       Q2.5    Q97.5
## Intercept              0.9764197 1.3323302 -1.6538552 3.635663
## ConditionFocusedevery 1.7436850 0.6829968  0.4776295 3.171128

draws <- as_draws_df(fit_full)
names(draws)[grepl("^b_", names(draws))]

## [1] "b_Intercept"             "b_ConditionFocusedevery"

beta <- draws[["b_ConditionFocusedevery"]]

mean(beta)

## [1] 1.743685
```

```r
quantile(beta, c(.025, .975))

##      2.5%     97.5%
## 0.4776295 3.1711280

bayestestR::hdi(beta, ci = 0.95)

## 95% HDI: [0.44, 3.13]

mean(beta > 0)

## [1] 0.9972875

# 13b. Probabilities for an AVERAGE subject and item
nd  <- data.frame(Condition = levels(Experiment1_only$Condition))
epred_avg <- posterior_epred(fit_full, newdata = nd, re_formula = NA)
colnames(epred_avg) <- nd$Condition

apply(epred_avg, 2, mean)

##      Baseline Focused every
##     0.6759661     0.8878826

apply(epred_avg, 2, function(x) bayestestR::hdi(x, ci = 0.95))

## $Baseline
## 95% HDI: [0.23, 1.00]
##
## $`Focused every`
## 95% HDI: [0.61, 1.00]

# 13c. Probabilities MARGINAL over the random effects
epred_marg <- posterior_epred(fit_full, newdata = nd, re_formula = NULL,
                             allow_new_levels = TRUE,
                             sample_new_levels = "gaussian")
colnames(epred_marg) <- nd$Condition
apply(epred_marg, 2, mean)

##      Baseline Focused every
##     0.5569615     0.6561264

apply(epred_marg, 2, function(x) bayestestR::hdi(x, ci = 0.95))

## $Baseline
## 95% HDI: [0.00, 1.00]
##
## $`Focused every`
## 95% HDI: [0.00, 1.00]

# 13d. The DIFFERENCE in probability
diff_p <- epred_avg[, 2] - epred_avg[, 1]
mean(diff_p)
```

```
## [1] 0.2119166

bayestestR::hdi(diff_p, ci = 0.95)

## 95% HDI: [0.00, 0.49]

mean(diff_p > 0)

## [1] 0.9972875

################################################################################
###
# STEP 13 — Bayes factor, with a stability check
################################################################################
###

set.seed(2024)
bf_reps <- replicate(5, {
  bayes_factor(fit_full, fit_null)$bf
})

## Iteration: 1
## Iteration: 2
## Iteration: 3
## Iteration: 4
## Iteration: 5
## Iteration: 6
## Iteration: 1
## Iteration: 2
## Iteration: 3
## Iteration: 4
## Iteration: 5
## Iteration: 6
## Iteration: 1
## Iteration: 2
## Iteration: 3
## Iteration: 4
## Iteration: 5
## Iteration: 6
## Iteration: 1
## Iteration: 2
## Iteration: 3
## Iteration: 4
## Iteration: 5
## Iteration: 6
## Iteration: 1
## Iteration: 2
## Iteration: 3
## Iteration: 4
## Iteration: 5
## Iteration: 1
```

```
## Iteration: 2
## Iteration: 3
## Iteration: 4
## Iteration: 5
## Iteration: 1
## Iteration: 2
## Iteration: 3
## Iteration: 4
## Iteration: 5
## Iteration: 6
## Iteration: 1
## Iteration: 2
## Iteration: 3
## Iteration: 4
## Iteration: 1
## Iteration: 2
## Iteration: 3
## Iteration: 4
## Iteration: 5
## Iteration: 1
## Iteration: 2
## Iteration: 3
## Iteration: 4
## Iteration: 5
## Iteration: 6
```

```
bf_reps
```

```
## [1] 13.92697 13.72793 13.67168 13.71522 13.51003
```

```
mean(bf_reps)
```

```
## [1] 13.71037
```

```
sd(bf_reps)
```

```
## [1] 0.149064
```

```
range(bf_reps)
```

```
## [1] 13.51003 13.92697
```

```
##############################################################################
###
# STEP 14 — Prior sensitivity analysis
##############################################################################
###

run_bf_for_sd <- function(sd_b, tag) {
  pf <- c(prior_string(paste0("normal(0, ", sd_b, ")"), class = "b"),
          prior_string(paste0("normal(0, ", sd_b, ")"), class = "b", coef =
"Intercept"),
```

```
          prior(student_t(3, 0, 2), class = "sd"))
  pn <- c(prior_string(paste0("normal(0, ", sd_b, ")"), class = "b", coef =
"Intercept"),
          prior(student_t(3, 0, 2), class = "sd"))

  ff <- brm(Accuracy ~ 0 + Intercept + Condition + (1 | Subject) + (1 |
Item),
            data = Experiment1_only, family = bernoulli(),
            prior = pf, chains = 4, iter = 25000, warmup = 5000,
            control = list(adapt_delta = 0.99), save_pars = save_pars(all =
TRUE),
            seed = 123, file = paste0("fits/full_sd", tag))

  fn <- brm(Accuracy ~ 0 + Intercept + (1 | Subject) + (1 | Item),
            data = Experiment1_only, family = bernoulli(),
            prior = pn, chains = 4, iter = 25000, warmup = 5000,
            control = list(adapt_delta = 0.99), save_pars = save_pars(all =
TRUE),
            seed = 123, file = paste0("fits/null_sd", tag))

  bayes_factor(ff, fn)$bf
}

bf_sd1 <- run_bf_for_sd(1,   "1")

## Compiling Stan program...
## Trying to compile a simple C file

## Running /Library/Frameworks/R.framework/Resources/bin/R CMD SHLIB foo.c
## using C compiler: ‘Apple clang version 16.0.0 (clang-1600.0.26.4)’
## using SDK: ‘MacOSX15.1.sdk’
## clang -arch arm64 -I"/Library/Frameworks/R.framework/Resources/include" -
DNDEBUG   -I"/Library/Frameworks/R.framework/Versions/4.4-
arm64/Resources/library/Rcpp/include/"  -
I"/Library/Frameworks/R.framework/Versions/4.4-
arm64/Resources/library/RcppEigen/include/"  -
I"/Library/Frameworks/R.framework/Versions/4.4-
arm64/Resources/library/RcppEigen/include/unsupported"  -
I"/Library/Frameworks/R.framework/Versions/4.4-
arm64/Resources/library/BH/include" -
I"/Library/Frameworks/R.framework/Versions/4.4-
arm64/Resources/library/StanHeaders/include/src/"  -
I"/Library/Frameworks/R.framework/Versions/4.4-
arm64/Resources/library/StanHeaders/include/"  -
I"/Library/Frameworks/R.framework/Versions/4.4-
arm64/Resources/library/RcppParallel/include/"  -
I"/Library/Frameworks/R.framework/Versions/4.4-
arm64/Resources/library/rstan/include" -DEIGEN_NO_DEBUG  -
DBOOST_DISABLE_ASSERTS  -DBOOST_PENDING_INTEGER_LOG2_HPP  -DSTAN_THREADS  -
DUSE_STANC3 -DSTRICT_R_HEADERS  -DBOOST_PHOENIX_NO_VARIADIC_EXPRESSION  -
```

```
D_HAS_AUTO_PTR_ETC=0  -include '/Library/Frameworks/R.framework/Versions/4.4-
arm64/Resources/library/StanHeaders/include/stan/math/prim/fun/Eigen.hpp'  -
D_REENTRANT -DRCPP_PARALLEL_USE_TBB=1   -I/opt/R/arm64/include    -fPIC  -
falign-functions=64 -Wall -g -O2  -c foo.c -o foo.o
## In file included from <built-in>:1:
## In file included from /Library/Frameworks/R.framework/Versions/4.4-
arm64/Resources/library/StanHeaders/include/stan/math/prim/fun/Eigen.hpp:22:
## In file included from /Library/Frameworks/R.framework/Versions/4.4-
arm64/Resources/library/RcppEigen/include/Eigen/Dense:1:
## In file included from /Library/Frameworks/R.framework/Versions/4.4-
arm64/Resources/library/RcppEigen/include/Eigen/Core:19:
## /Library/Frameworks/R.framework/Versions/4.4-
arm64/Resources/library/RcppEigen/include/Eigen/src/Core/util/Macros.h:679:10
: fatal error: 'cmath' file not found
##   679 | #include <cmath>
##       |          ^~~~~~~
## 1 error generated.
## make: *** [foo.o] Error 1

## Start sampling

## Compiling Stan program...

## Trying to compile a simple C file

## Running /Library/Frameworks/R.framework/Resources/bin/R CMD SHLIB foo.c
## using C compiler: ‘Apple clang version 16.0.0 (clang-1600.0.26.4)’
## using SDK: ‘MacOSX15.1.sdk’
## clang -arch arm64 -I"/Library/Frameworks/R.framework/Resources/include" -
DNDEBUG   -I"/Library/Frameworks/R.framework/Versions/4.4-
arm64/Resources/library/Rcpp/include/"  -
I"/Library/Frameworks/R.framework/Versions/4.4-
arm64/Resources/library/RcppEigen/include/"  -
I"/Library/Frameworks/R.framework/Versions/4.4-
arm64/Resources/library/RcppEigen/include/unsupported"  -
I"/Library/Frameworks/R.framework/Versions/4.4-
arm64/Resources/library/BH/include" -
I"/Library/Frameworks/R.framework/Versions/4.4-
arm64/Resources/library/StanHeaders/include/src/"  -
I"/Library/Frameworks/R.framework/Versions/4.4-
arm64/Resources/library/StanHeaders/include/"  -
I"/Library/Frameworks/R.framework/Versions/4.4-
arm64/Resources/library/RcppParallel/include/"  -
I"/Library/Frameworks/R.framework/Versions/4.4-
arm64/Resources/library/rstan/include" -DEIGEN_NO_DEBUG  -
DBOOST_DISABLE_ASSERTS  -DBOOST_PENDING_INTEGER_LOG2_HPP  -DSTAN_THREADS  -
DUSE_STANC3 -DSTRICT_R_HEADERS  -DBOOST_PHOENIX_NO_VARIADIC_EXPRESSION  -
D_HAS_AUTO_PTR_ETC=0  -include '/Library/Frameworks/R.framework/Versions/4.4-
arm64/Resources/library/StanHeaders/include/stan/math/prim/fun/Eigen.hpp'  -
D_REENTRANT -DRCPP_PARALLEL_USE_TBB=1   -I/opt/R/arm64/include    -fPIC  -
falign-functions=64 -Wall -g -O2  -c foo.c -o foo.o
```

```
## In file included from <built-in>:1:
## In file included from /Library/Frameworks/R.framework/Versions/4.4-
arm64/Resources/library/StanHeaders/include/stan/math/prim/fun/Eigen.hpp:22:
## In file included from /Library/Frameworks/R.framework/Versions/4.4-
arm64/Resources/library/RcppEigen/include/Eigen/Dense:1:
## In file included from /Library/Frameworks/R.framework/Versions/4.4-
arm64/Resources/library/RcppEigen/include/Eigen/Core:19:
## /Library/Frameworks/R.framework/Versions/4.4-
arm64/Resources/library/RcppEigen/include/Eigen/src/Core/util/Macros.h:679:10
: fatal error: 'cmath' file not found
##   679 | #include <cmath>
##       |          ^~~~~~~
## 1 error generated.
## make: *** [foo.o] Error 1

## Start sampling

## Iteration: 1
## Iteration: 2
## Iteration: 3
## Iteration: 4
## Iteration: 5
## Iteration: 1
## Iteration: 2
## Iteration: 3
## Iteration: 4
## Iteration: 5

bf_sd3 <- run_bf_for_sd(3,   "3")

## Compiling Stan program...
## Trying to compile a simple C file

## Running /Library/Frameworks/R.framework/Resources/bin/R CMD SHLIB foo.c
## using C compiler: ‘Apple clang version 16.0.0 (clang-1600.0.26.4)’
## using SDK: ‘MacOSX15.1.sdk’
## clang -arch arm64 -I"/Library/Frameworks/R.framework/Resources/include" -
DNDEBUG   -I"/Library/Frameworks/R.framework/Versions/4.4-
arm64/Resources/library/Rcpp/include/"  -
I"/Library/Frameworks/R.framework/Versions/4.4-
arm64/Resources/library/RcppEigen/include/"  -
I"/Library/Frameworks/R.framework/Versions/4.4-
arm64/Resources/library/RcppEigen/include/unsupported"  -
I"/Library/Frameworks/R.framework/Versions/4.4-
arm64/Resources/library/BH/include" -
I"/Library/Frameworks/R.framework/Versions/4.4-
arm64/Resources/library/StanHeaders/include/src/"  -
I"/Library/Frameworks/R.framework/Versions/4.4-
arm64/Resources/library/StanHeaders/include/"  -
I"/Library/Frameworks/R.framework/Versions/4.4-
arm64/Resources/library/RcppParallel/include/"  -
```

```
I"/Library/Frameworks/R.framework/Versions/4.4-
arm64/Resources/library/rstan/include" -DEIGEN_NO_DEBUG  -
DBOOST_DISABLE_ASSERTS  -DBOOST_PENDING_INTEGER_LOG2_HPP  -DSTAN_THREADS  -
DUSE_STANC3 -DSTRICT_R_HEADERS  -DBOOST_PHOENIX_NO_VARIADIC_EXPRESSION  -
D_HAS_AUTO_PTR_ETC=0  -include '/Library/Frameworks/R.framework/Versions/4.4-
arm64/Resources/library/StanHeaders/include/stan/math/prim/fun/Eigen.hpp'  -
D_REENTRANT -DRCPP_PARALLEL_USE_TBB=1   -I/opt/R/arm64/include    -fPIC  -
falign-functions=64 -Wall -g -O2  -c foo.c -o foo.o
## In file included from <built-in>:1:
## In file included from /Library/Frameworks/R.framework/Versions/4.4-
arm64/Resources/library/StanHeaders/include/stan/math/prim/fun/Eigen.hpp:22:
## In file included from /Library/Frameworks/R.framework/Versions/4.4-
arm64/Resources/library/RcppEigen/include/Eigen/Dense:1:
## In file included from /Library/Frameworks/R.framework/Versions/4.4-
arm64/Resources/library/RcppEigen/include/Eigen/Core:19:
## /Library/Frameworks/R.framework/Versions/4.4-
arm64/Resources/library/RcppEigen/include/Eigen/src/Core/util/Macros.h:679:10
: fatal error: 'cmath' file not found
##   679 | #include <cmath>
##       |          ^~~~~~~
## 1 error generated.
## make: *** [foo.o] Error 1

## Start sampling

## Compiling Stan program...

## Trying to compile a simple C file

## Running /Library/Frameworks/R.framework/Resources/bin/R CMD SHLIB foo.c
## using C compiler: ‘Apple clang version 16.0.0 (clang-1600.0.26.4)’
## using SDK: ‘MacOSX15.1.sdk’
## clang -arch arm64 -I"/Library/Frameworks/R.framework/Resources/include" -
DNDEBUG   -I"/Library/Frameworks/R.framework/Versions/4.4-
arm64/Resources/library/Rcpp/include/"  -
I"/Library/Frameworks/R.framework/Versions/4.4-
arm64/Resources/library/RcppEigen/include/"  -
I"/Library/Frameworks/R.framework/Versions/4.4-
arm64/Resources/library/RcppEigen/include/unsupported"  -
I"/Library/Frameworks/R.framework/Versions/4.4-
arm64/Resources/library/BH/include" -
I"/Library/Frameworks/R.framework/Versions/4.4-
arm64/Resources/library/StanHeaders/include/src/"  -
I"/Library/Frameworks/R.framework/Versions/4.4-
arm64/Resources/library/StanHeaders/include/"  -
I"/Library/Frameworks/R.framework/Versions/4.4-
arm64/Resources/library/RcppParallel/include/"  -
I"/Library/Frameworks/R.framework/Versions/4.4-
arm64/Resources/library/rstan/include" -DEIGEN_NO_DEBUG  -
DBOOST_DISABLE_ASSERTS  -DBOOST_PENDING_INTEGER_LOG2_HPP  -DSTAN_THREADS  -
DUSE_STANC3 -DSTRICT_R_HEADERS  -DBOOST_PHOENIX_NO_VARIADIC_EXPRESSION  -
```

```
D_HAS_AUTO_PTR_ETC=0  -include '/Library/Frameworks/R.framework/Versions/4.4-arm64/Resources/library/StanHeaders/include/stan/math/prim/fun/Eigen.hpp'  -D_REENTRANT -DRCPP_PARALLEL_USE_TBB=1   -I/opt/R/arm64/include    -fPIC  -falign-functions=64 -Wall -g -O2  -c foo.c -o foo.o
## In file included from <built-in>:1:
## In file included from /Library/Frameworks/R.framework/Versions/4.4-arm64/Resources/library/StanHeaders/include/stan/math/prim/fun/Eigen.hpp:22:
## In file included from /Library/Frameworks/R.framework/Versions/4.4-arm64/Resources/library/RcppEigen/include/Eigen/Dense:1:
## In file included from /Library/Frameworks/R.framework/Versions/4.4-arm64/Resources/library/RcppEigen/include/Eigen/Core:19:
## /Library/Frameworks/R.framework/Versions/4.4-arm64/Resources/library/RcppEigen/include/Eigen/src/Core/util/Macros.h:679:10: fatal error: 'cmath' file not found
##   679 | #include <cmath>
##       |          ^~~~~~~
## 1 error generated.
## make: *** [foo.o] Error 1

## Start sampling

## Iteration: 1
## Iteration: 2
## Iteration: 3
## Iteration: 4
## Iteration: 5
## Iteration: 1
## Iteration: 2
## Iteration: 3
## Iteration: 4
## Iteration: 5
## Iteration: 6

data.frame(prior_sd = c(1, 2, 3),
           BF10     = c(bf_sd1, mean(bf_reps), bf_sd3))

##   prior_sd     BF10
## 1        1 11.74932
## 2        2 13.71037
## 3        3 11.05262

###############################################################################
# STEP 15 — Maximal random-effects structure (robustness check)
###############################################################################

# 16a. glmer
m_slope_ml <- try(
  glmer(Accuracy ~ Condition + (1 + Condition | Subject) + (1 | Item),
        data = Experiment1_only, family = binomial(link = "logit"),
```

```
        control = glmerControl(optimizer = "bobyqa")),
  silent = TRUE
)
if (!inherits(m_slope_ml, "try-error")) {
  print(isSingular(m_slope_ml))
  print(VarCorr(m_slope_ml), comp = "Std.Dev.")
}

## [1] FALSE
##  Groups   Name                   Std.Dev. Corr
##  Subject (Intercept)             15.4868
##          ConditionFocused every  3.7803  -0.873
##  Item    (Intercept)              1.0229

# 16b. brms
priors_slope <- c(
  prior(normal(0, 2),       class = "b"),
  prior(normal(0, 2),       class = "b", coef = "Intercept"),
  prior(student_t(3, 0, 2), class = "sd"),
  prior(lkj(2),             class = "cor")
)

fit_slope <- brm(
  Accuracy ~ 0 + Intercept + Condition + (1 + Condition | Subject) + (1 |
Item),
  data      = Experiment1_only,
  family    = bernoulli(link = "logit"),
  prior     = priors_slope,
  chains    = 4, iter = 15000, warmup = 5000,
  control   = list(adapt_delta = 0.99, max_treedepth = 12),
  seed      = 123,
  file      = "fits/fit_slope"
)

## Compiling Stan program...
## Trying to compile a simple C file

## Running /Library/Frameworks/R.framework/Resources/bin/R CMD SHLIB foo.c
## using C compiler: ‘Apple clang version 16.0.0 (clang-1600.0.26.4)’
## using SDK: ‘MacOSX15.1.sdk’
## clang -arch arm64 -I"/Library/Frameworks/R.framework/Resources/include" -
DNDEBUG   -I"/Library/Frameworks/R.framework/Versions/4.4-
arm64/Resources/library/Rcpp/include/"  -
I"/Library/Frameworks/R.framework/Versions/4.4-
arm64/Resources/library/RcppEigen/include/"  -
I"/Library/Frameworks/R.framework/Versions/4.4-
arm64/Resources/library/RcppEigen/include/unsupported"  -
I"/Library/Frameworks/R.framework/Versions/4.4-
arm64/Resources/library/BH/include" -
I"/Library/Frameworks/R.framework/Versions/4.4-
arm64/Resources/library/StanHeaders/include/src/"  -
```

```
I"/Library/Frameworks/R.framework/Versions/4.4-
arm64/Resources/library/StanHeaders/include/"  -
I"/Library/Frameworks/R.framework/Versions/4.4-
arm64/Resources/library/RcppParallel/include/"  -
I"/Library/Frameworks/R.framework/Versions/4.4-
arm64/Resources/library/rstan/include" -DEIGEN_NO_DEBUG  -
DBOOST_DISABLE_ASSERTS  -DBOOST_PENDING_INTEGER_LOG2_HPP  -DSTAN_THREADS  -
DUSE_STANC3 -DSTRICT_R_HEADERS  -DBOOST_PHOENIX_NO_VARIADIC_EXPRESSION  -
D_HAS_AUTO_PTR_ETC=0  -include '/Library/Frameworks/R.framework/Versions/4.4-
arm64/Resources/library/StanHeaders/include/stan/math/prim/fun/Eigen.hpp'  -
D_REENTRANT -DRCPP_PARALLEL_USE_TBB=1   -I/opt/R/arm64/include    -fPIC  -
falign-functions=64 -Wall -g -O2  -c foo.c -o foo.o
## In file included from <built-in>:1:
## In file included from /Library/Frameworks/R.framework/Versions/4.4-
arm64/Resources/library/StanHeaders/include/stan/math/prim/fun/Eigen.hpp:22:
## In file included from /Library/Frameworks/R.framework/Versions/4.4-
arm64/Resources/library/RcppEigen/include/Eigen/Dense:1:
## In file included from /Library/Frameworks/R.framework/Versions/4.4-
arm64/Resources/library/RcppEigen/include/Eigen/Core:19:
## /Library/Frameworks/R.framework/Versions/4.4-
arm64/Resources/library/RcppEigen/include/Eigen/src/Core/util/Macros.h:679:10
: fatal error: 'cmath' file not found
##   679 | #include <cmath>
##       |          ^~~~~~~
## 1 error generated.
## make: *** [foo.o] Error 1

## Start sampling
```

```
summary(fit_slope)
```

```
##  Family: bernoulli
##   Links: mu = logit
## Formula: Accuracy ~ 0 + Intercept + Condition + (1 + Condition | Subject)
+ (1 | Item)
##    Data: Experiment1_only (Number of observations: 180)
##   Draws: 4 chains, each with iter = 15000; warmup = 5000; thin = 1;
##          total post-warmup draws = 40000
##
## Multilevel Hyperparameters:
## ~Item (Number of levels: 5)
##               Estimate Est.Error l-95% CI u-95% CI Rhat Bulk_ESS Tail_ESS
## sd(Intercept)     1.04      0.71     0.08     2.79 1.00    15075    15317
##
## ~Subject (Number of levels: 18)
##                                        Estimate Est.Error l-95% CI u-95% CI
Rhat
## sd(Intercept)                              6.85      2.38     3.58    12.73
1.00
## sd(ConditionFocusedevery)                  1.46      1.09     0.06     4.06
```

```
1.00
## cor(Intercept,ConditionFocusedevery)     0.05      0.44    -0.78     0.82
1.00
##                                      Bulk_ESS Tail_ESS
## sd(Intercept)                           17936    25661
## sd(ConditionFocusedevery)               18338    17655
## cor(Intercept,ConditionFocusedevery)    49630    27696
##
## Regression Coefficients:
##                         Estimate Est.Error l-95% CI u-95% CI Rhat Bulk_ESS
## Intercept                   0.98      1.37    -1.71     3.69 1.00    14914
## ConditionFocusedevery       1.85      0.88     0.20     3.71 1.00    38733
##                         Tail_ESS
## Intercept                  22513
## ConditionFocusedevery      26021
##
## Draws were sampled using sampling(NUTS). For each parameter, Bulk_ESS
## and Tail_ESS are effective sample size measures, and Rhat is the potential
## scale reduction factor on split chains (at convergence, Rhat = 1).

fixef(fit_slope)

##                          Estimate Est.Error       Q2.5    Q97.5
## Intercept               0.9818822 1.3660944 -1.7102229 3.687418
## ConditionFocusedevery 1.8499890 0.8820761  0.2027855 3.712017

###############################################################################
###
# STEP 16 — Age model (exploratory)
###############################################################################
###

Experiment1_only$Age_c <- Experiment1_only$Age - mean(Experiment1_only$Age)

fit_age <- brm(
  Accuracy ~ 0 + Intercept + Condition * Age_c + (1 | Subject) + (1 | Item),
  data    = Experiment1_only,
  family  = bernoulli(link = "logit"),
  prior   = c(prior(normal(0, 2), class = "b"),
              prior(student_t(3, 0, 2), class = "sd")),
  chains  = 4, iter = 10000, warmup = 2000,
  control = list(adapt_delta = 0.99),
  seed    = 123,
  file    = "fits/fit_age"
)

## Compiling Stan program...
## Trying to compile a simple C file

## Running /Library/Frameworks/R.framework/Resources/bin/R CMD SHLIB foo.c
## using C compiler: ‘Apple clang version 16.0.0 (clang-1600.0.26.4)’
```

```
## using SDK: ‘MacOSX15.1.sdk’
## clang -arch arm64 -I"/Library/Frameworks/R.framework/Resources/include" -
DNDEBUG   -I"/Library/Frameworks/R.framework/Versions/4.4-
arm64/Resources/library/Rcpp/include/"  -
I"/Library/Frameworks/R.framework/Versions/4.4-
arm64/Resources/library/RcppEigen/include/"  -
I"/Library/Frameworks/R.framework/Versions/4.4-
arm64/Resources/library/RcppEigen/include/unsupported"  -
I"/Library/Frameworks/R.framework/Versions/4.4-
arm64/Resources/library/BH/include" -
I"/Library/Frameworks/R.framework/Versions/4.4-
arm64/Resources/library/StanHeaders/include/src/"  -
I"/Library/Frameworks/R.framework/Versions/4.4-
arm64/Resources/library/StanHeaders/include/"  -
I"/Library/Frameworks/R.framework/Versions/4.4-
arm64/Resources/library/RcppParallel/include/"  -
I"/Library/Frameworks/R.framework/Versions/4.4-
arm64/Resources/library/rstan/include" -DEIGEN_NO_DEBUG  -
DBOOST_DISABLE_ASSERTS  -DBOOST_PENDING_INTEGER_LOG2_HPP  -DSTAN_THREADS  -
DUSE_STANC3 -DSTRICT_R_HEADERS  -DBOOST_PHOENIX_NO_VARIADIC_EXPRESSION  -
D_HAS_AUTO_PTR_ETC=0  -include '/Library/Frameworks/R.framework/Versions/4.4-
arm64/Resources/library/StanHeaders/include/stan/math/prim/fun/Eigen.hpp'  -
D_REENTRANT -DRCPP_PARALLEL_USE_TBB=1   -I/opt/R/arm64/include    -fPIC  -
falign-functions=64 -Wall -g -O2  -c foo.c -o foo.o
## In file included from <built-in>:1:
## In file included from /Library/Frameworks/R.framework/Versions/4.4-
arm64/Resources/library/StanHeaders/include/stan/math/prim/fun/Eigen.hpp:22:
## In file included from /Library/Frameworks/R.framework/Versions/4.4-
arm64/Resources/library/RcppEigen/include/Eigen/Dense:1:
## In file included from /Library/Frameworks/R.framework/Versions/4.4-
arm64/Resources/library/RcppEigen/include/Eigen/Core:19:
## /Library/Frameworks/R.framework/Versions/4.4-
arm64/Resources/library/RcppEigen/include/Eigen/src/Core/util/Macros.h:679:10
: fatal error: 'cmath' file not found
##   679 | #include <cmath>
##       |          ^~~~~~~
## 1 error generated.
## make: *** [foo.o] Error 1

## Start sampling
```

```
summary(fit_age)
```

```
##  Family: bernoulli
##   Links: mu = logit
## Formula: Accuracy ~ 0 + Intercept + Condition * Age_c + (1 | Subject) + (1
| Item)
##    Data: Experiment1_only (Number of observations: 180)
##   Draws: 4 chains, each with iter = 10000; warmup = 2000; thin = 1;
##          total post-warmup draws = 32000
```

```
##
## Multilevel Hyperparameters:
## ~Item (Number of levels: 5)
##               Estimate Est.Error l-95% CI u-95% CI Rhat Bulk_ESS Tail_ESS
## sd(Intercept)     1.04      0.72     0.07     2.77 1.00     9633    10309
##
## ~Subject (Number of levels: 18)
##               Estimate Est.Error l-95% CI u-95% CI Rhat Bulk_ESS Tail_ESS
## sd(Intercept)     7.06      2.40     3.77    12.97 1.00    10771    17064
##
## Regression Coefficients:
##                             Estimate Est.Error l-95% CI u-95% CI Rhat
Bulk_ESS
## Intercept                       0.94      1.38    -1.73     3.69 1.00
8937
## ConditionFocusedevery           2.01      0.74     0.66     3.55 1.00
25500
## Age_c                          -0.35      1.69    -3.66     3.04 1.00
13245
## ConditionFocusedevery:Age_c     1.35      1.15    -0.84     3.69 1.00
24914
##                             Tail_ESS
## Intercept                      14407
## ConditionFocusedevery          20848
## Age_c                          18641
## ConditionFocusedevery:Age_c    21518
##
## Draws were sampled using sampling(NUTS). For each parameter, Bulk_ESS
## and Tail_ESS are effective sample size measures, and Rhat is the potential
## scale reduction factor on split chains (at convergence, Rhat = 1).


#############################################################################
###
# STEP 17 — Reproducibility record
#############################################################################
###

sessionInfo()

## R version 4.4.0 (2024-04-24)
## Platform: aarch64-apple-darwin20
## Running under: macOS 26.5
##
## Matrix products: default
## BLAS:   /Library/Frameworks/R.framework/Versions/4.4-
arm64/Resources/lib/libRblas.0.dylib
## LAPACK: /Library/Frameworks/R.framework/Versions/4.4-
arm64/Resources/lib/libRlapack.dylib;  LAPACK version 3.12.0
##
```

```
## locale:
## [1] en_US.UTF-8/en_US.UTF-8/en_US.UTF-8/C/en_US.UTF-8/en_US.UTF-8
##
## time zone: Europe/Istanbul
## tzcode source: internal
##
## attached base packages:
## [1] stats     graphics  grDevices utils     datasets  methods   base
##
## other attached packages:
## [1] posterior_1.6.0   bayestestR_0.17.0 brms_2.21.0       Rcpp_1.0.12
## [5] emmeans_1.10.2    lme4_1.1-35.3     Matrix_1.7-0      ggplot2_3.5.1
## [9] dplyr_1.1.4
##
## loaded via a namespace (and not attached):
##  [1] tidyselect_1.2.1     farver_2.1.2         loo_2.8.0
##  [4] fastmap_1.2.0        TH.data_1.1-2        tensorA_0.36.2.1
##  [7] digest_0.6.35        estimability_1.5.1   lifecycle_1.0.4
## [10] StanHeaders_2.32.10  survival_3.5-8       processx_3.8.4
## [13] magrittr_2.0.3       compiler_4.4.0       rlang_1.1.6
## [16] tools_4.4.0          utf8_1.2.4           yaml_2.3.8
## [19] knitr_1.46           labeling_0.4.3       bridgesampling_1.1-2
## [22] pkgbuild_1.4.4       plyr_1.8.9           RColorBrewer_1.1-3
## [25] multcomp_1.4-25      abind_1.4-5          withr_3.0.0
## [28] purrr_1.2.0          grid_4.4.0           stats4_4.4.0
## [31] fansi_1.0.6          xtable_1.8-4         colorspace_2.1-0
## [34] inline_0.3.19        scales_1.4.0         MASS_7.3-60.2
## [37] insight_1.4.6        cli_3.6.5            mvtnorm_1.2-5
## [40] rmarkdown_2.27       ragg_1.3.2           generics_0.1.3
## [43] RcppParallel_5.1.9   rstudioapi_0.17.1    reshape2_1.4.4
## [46] minqa_1.2.6          rstan_2.32.6         stringr_1.5.1
## [49] splines_4.4.0        bayesplot_1.11.1     parallel_4.4.0
## [52] matrixStats_1.4.1    vctrs_0.6.5          boot_1.3-30
## [55] sandwich_3.1-0       callr_3.7.6          systemfonts_1.1.0
## [58] tidyr_1.3.1          glue_1.7.0           nloptr_2.0.3
## [61] codetools_0.2-20     ps_1.7.6             distributional_0.5.0
## [64] stringi_1.8.4        gtable_0.3.6         QuickJSR_1.3.1
## [67] tibble_3.2.1         pillar_1.9.0         htmltools_0.5.8.1
## [70] Brobdingnag_1.2-9    R6_2.5.1             textshaping_0.4.0
## [73] evaluate_0.23        lattice_0.22-6       backports_1.5.0
## [76] rstantools_2.4.0     coda_0.19-4.1        gridExtra_2.3
## [79] nlme_3.1-164         checkmate_2.3.2      xfun_0.52
## [82] zoo_1.8-12           pkgconfig_2.0.3
```

```
citation("lme4"); citation("brms"); citation("emmeans");
citation("bridgesampling")
```

```
## To cite lme4 in publications use:
##
##   Douglas Bates, Martin Maechler, Ben Bolker, Steve Walker (2015).
```

```
##   Fitting Linear Mixed-Effects Models Using lme4. Journal of
##   Statistical Software, 67(1), 1-48. doi:10.18637/jss.v067.i01.
##
## A BibTeX entry for LaTeX users is
##
##   @Article{,
##     title = {Fitting Linear Mixed-Effects Models Using {lme4}},
##     author = {Douglas Bates and Martin M{\"a}chler and Ben Bolker and
Steve Walker},
##     journal = {Journal of Statistical Software},
##     year = {2015},
##     volume = {67},
##     number = {1},
##     pages = {1--48},
##     doi = {10.18637/jss.v067.i01},
##   }

## To cite brms in publications use:
##
##   Paul-Christian Bürkner (2017). brms: An R Package for Bayesian
##   Multilevel Models Using Stan. Journal of Statistical Software, 80(1),
##   1-28. doi:10.18637/jss.v080.i01
##
## Paul-Christian Bürkner (2018). Advanced Bayesian Multilevel Modeling
## with the R Package brms. The R Journal, 10(1), 395-411.
## doi:10.32614/RJ-2018-017
##
## Paul-Christian Bürkner (2021). Bayesian Item Response Modeling in R
## with brms and Stan. Journal of Statistical Software, 100(5), 1-54.
## doi:10.18637/jss.v100.i05
##
## To see these entries in BibTeX format, use 'print(<citation>,
## bibtex=TRUE)', 'toBibtex(.)', or set
## 'options(citation.bibtex.max=999)'.

## To cite package 'emmeans' in publications use:
##
##   Lenth R (2024). _emmeans: Estimated Marginal Means, aka Least-Squares
##   Means_. R package version 1.10.2,
##   <https://CRAN.R-project.org/package=emmeans>.
##
## A BibTeX entry for LaTeX users is
##
##   @Manual{,
##     title = {emmeans: Estimated Marginal Means, aka Least-Squares Means},
##     author = {Russell V. Lenth},
##     year = {2024},
##     note = {R package version 1.10.2},
##     url = {https://CRAN.R-project.org/package=emmeans},
##   }
```

```
## To cite bridgesampling in publications use:
##
##   Gronau QF, Singmann H, Wagenmakers E (2020). "bridgesampling: An R
##   Package for Estimating Normalizing Constants." _Journal of
##   Statistical Software_, *92*(10), 1-29. doi:10.18637/jss.v092.i10
##   <https://doi.org/10.18637/jss.v092.i10>.
##
## A BibTeX entry for LaTeX users is
##
##   @Article{,
##     title = {{bridgesampling}: An {R} Package for Estimating Normalizing
Constants},
##     author = {Quentin F. Gronau and Henrik Singmann and Eric-Jan
Wagenmakers},
##     journal = {Journal of Statistical Software},
##     year = {2020},
##     volume = {92},
##     number = {10},
##     pages = {1--29},
##     doi = {10.18637/jss.v092.i10},
##   }

#############################################################################
###
```
