# Supplementary material for: Examining children’s sensitivity to morphologically-marked focus in the comprehension of question sentences: evidence from quantifier comprehension in child Turkish
Source: Front Hum Neurosci. 2026 Oct 14;20:1914200. doi: 10.3389/fnhum.2026.1914200 (PMC13714967; doi:10.3389/fnhum.2026.1914200)
Supplement: Supplementary file 2 [file Supplementary_file_1.pdf]

Supplementary Material

Appendix A:

<u>Experiment 1 materials:</u>

Focused *every* condition: Experimental items

(1)

Her	çocuk	mu	bir	balon	tut-uyor?
every	child	QP	a	balloonhold-PROG	

'Is it every child that is holding a balloon?'

(2)

Her	çocuk	mu	bir	araba	sür-üyor?
every	child	QP	a	car	drive-PROG

'Is it every child that is driving a car?'

(3)

Her	çocuk	mu	bir	kitap	ok-uyor?
every	child	QP	a	book	read-PROG

'Is it every child that is reading a book?'

(4)

Her	çocuk	mu	bir	gitar	çal-ıyor?
every	child	QP	a	guitar	play-PROG

'Is it every child that is playing a guitar?'

(5)

Her	çocuk	mu	bir	kutu	taş-ıyor?
every	child	QP	a	box	carry-PROG

'Is it every child that is carrying a box?'

Focused *every* condition: Filler items

(1)

Her	kedi	mi	uy-uyor?
every	cat	QP	sleep-PROG

‘Is it every cat that is sleeping?’

(2)

Her	maymun	mu	şapka	tak-ıyor?
every	monkey	QP	hat	wear-PROG

‘Is it every monkey that is wearing a hat?’

(3)

Her	köpek	mi	kahverengi	tüylü?
every	dog	QP	brown	fur

‘Is it every dog that has a brown fur?’

(4)

Her	kurbağa	mı	turuncu?
every	frog	QP	orange

‘Is it every frog that is orange?’

(5)

Her	kaplumbağa	mı	su-da	yüz-üyor?
every	turtle	QP	water-LOC	swim-PROG

‘Is it every turtle that is swimming in the water?’

Baseline condition: Experimental items

(1) Her çocuk bir balon tut-uyor mu?
every child a balloonhold-PROG QP
'Is every child holding a balloon?'

(2) Her çocuk bir araba sür-üyor mu?
every child a car drive-PROG QP
'Is every child driving a car?'

(3) Her çocuk bir kitap ok-uyor mu?
every child a book read-PROG QP
'Is every child reading a book?'

(4) Her çocuk bir kitap ok-uyor mu?
every child a book read-PROG QP
'Is every child reading a book?'

(5) Her çocuk bir gitar çal-ıyor mu?
every child a guitar play-PROG QP
'Is every child playing a guitar?'

Baseline condition: Filler items

(1)

Her	kedi	uy-uyor		mu?
every	cat	sleep-PROG		QP

'Is every cat sleeping?'

(2)

Her	maymun	şapka	tak-ıyor	mu?
every	monkey	hat	wear-PROG	QP

'Is every monkey wearing a hat?'

(3)

Her	köpek	kahverengi	tüylü	mü?
every	dog	brown	fur	QP

'Does every dog have a brown fur?'

(4)

Her	kurbağa	turuncu	mu?
every	frog	orange	QP

'Is every frog orange?'

(5)

Her	kaplumbağa	su-da	yüz-üyor	mu?
every	turtle	water-LOC	swim-PROG	QP

'Is every turtle swimming in the water?'
